# SNR and Standard Deviation of cGNSS-R and iGNSS-R Scatterometric Measurements

**DOI:** 10.3390/s17010183

**Published:** 2017-01-19

**Authors:** Alberto Alonso-Arroyo, Jorge Querol, Carlos Lopez-Martinez, Valery U. Zavorotny, Hyuk Park, Daniel Pascual, Raul Onrubia, Adriano Camps

**Affiliations:** 1 Department of Signal Theory and Communications, Universitat Politécnica de Catalunya—BarcelonaTech (UPC), 08034 Barcelona, Spain; jorge.querol@tsc.upc.edu (J.Q.); carlos.lopez@tsc.upc.edu (C.L.-M.); park.hyuk@tsc.upc.edu (H.P.); daniel.pascual@tsc.upc.edu (D.P.); onrubia@tsc.upc.edu (R.O.); 2 Earth System Research Laboratory (ERSL), National Oceanic and Atmospheric Administration (NOAA), Boulder, CO 80305-3337, USA; valery.zavorotny@noaa.gov

**Keywords:** SNR, cGNSS-R, iGNSS-R, GNSS-R, GNSS-Scatterometry

## Abstract

This work addresses the accuracy of the Global Navigation Satellite Systems (GNSS)-Reflectometry (GNSS-R) scatterometric measurements considering the presence of both coherent and incoherent scattered components, for both conventional GNSS-R (cGNSS-R) and interferometric GNSS-R (iGNSS-R) techniques. The coherent component is present for some type of surfaces, and it has been neglected until now because it vanishes for the sea surface scattering case. Taking into account the presence of both scattering components, the estimated Signal-to-Noise Ratio (SNR) for both techniques is computed based on the detectability criterion, as it is done in conventional GNSS applications. The non-coherent averaging operation is considered from a general point of view, taking into account that thermal noise contributions can be reduced by an extra factor of 0.88 dB when using partially overlapped or partially correlated samples. After the SNRs are derived, the received waveform’s peak variability is computed, which determines the system’s capability to measure geophysical parameters. This theoretical derivations are applied to the United Kingdom (UK) TechDemoSat-1 (UK TDS-1) and to the future GNSS REflectometry, Radio Occultation and Scatterometry on board the International Space Station (ISS) (GEROS-ISS) scenarios, in order to estimate the expected scatterometric performance of both missions.

## 1. Introduction

The analysis of the SNR is very important to determine the variance of the radar cross section or reflectivity, and therefore, to assess the system’s capability and accuracy to measure geophysical parameters. The accuracy of radar scatterometric measurements from space was first addressed in [1], where two different effects were analyzed: the bias due to a lack of precise knowledge of the exact value of the system’s parameters, and the random fluctuations of the measured signal. While the bias can be compensated for through appropriate instrument calibration, the random fluctuations of the signal (*speckle* noise [2]) can only be reduced by averaging. In [1], it was considered that both the signal and the system noise follow Gaussian statistics, that is, the received signal is purely incoherent.

GNSS-R is a field that emerged in 1988 with the proposal of the multi-static GNSS-based scatterometry technique for remote sensing [3]. In 1993, the PAssive Reflectometry and Interferometry System (PARIS) concept was proposed in order to do mesoscale altimetric measurements using the signals of opportunity provided by the GNSS satellites [4]. Due to the forward scattering geometry and the surface scattering properties, the reflected signal may not always obey the Gaussian statistics assumed in [1]. In such cases, the statistics of the reflected field follow a Hoyt distribution, which describes a Gaussian field plus a coherent component [5]. In that scenario, a new scatterometric analysis must be performed in order to estimate the scatterometric accuracy of GNSS-R techniques.

The first GNSS-R scatterometric analysis was performed in 2001 [6], where a statistical analysis of the scatterometric SNR was presented, determining the accuracy of the surface height retrieval and the minimum number of samples required to estimate wind speed from a space-borne platform using cGNSS-R. Therein, the power spectrum of the sea-surface-reflected waveform was also introduced, which is of high importance in this analysis. However, this spectrum was derived assuming that there was no coherent component, which validates the expression only for rough sea surfaces. In 2004 and 2006, a detailed study regarding the correlation function of the sea surface GNSS-R waveform was presented, including a stochastic voltage model of the reflected waveforms and their autocorrelation functions [7,8]. In 2011, the scatterometric accuracy of a PARIS-like instrument using the iGNSS-R approach was presented [9]. In all those studies, a Gaussian model like the one in [1] was assumed, because experimental evidence had confirmed that the sea-surface scattered signals follow complex Gaussian statistics.

The new data obtained from the UK TDS-1 satellite have shown that while the Gaussian statistics is a valid model for the sea surface scattered signals, it is not satisfactory for surfaces such as flat land areas, and in particular wetlands, sea-ice, and lakes [10]. For such surfaces the retrieved Delay-Doppler Map (DDM)s show a “K-shape” feature, as shown in [11], which indicates a presence of the coherent component, requiring the use of a Hoyt distribution to describe the statistics of the scattered signals. Following this evidence, this work extends the scatterometric analysis performed in [9] to the cGNSS-R case, and includes the presence of the coherent component in the scattered signals that was not considered in previous works.

This paper starts with a definition of the signal model for both cGNSS-R and iGNSS-R cases. Then, the correlation peak statistics, which is the interesting one in the scatterometric mode, is analyzed for the GNSS case. Subsequently the same analysis is performed to the GNSS-R case, including the effect of non-coherent integration. Later, the variability of the correlation peak is computed. Simulations analyzing the performance of the UK TDS-1 and GEROS-ISS mission in terms of SNR and correlation peak variability are performed. This paper ends with a discussion and a concluding section highlighting the main achievements.

## 2. Signal Model

Generally, two different approaches are used to process GNSS reflected signals. Initially, the iGNSS-R technique was proposed in [4], which consists of the cross-correlation of the reflected and direct signals which allows to use the entire signal bandwidth, including the encrypted codes that present a wider spectrum. The second approach proposed was the cGNSS-R technique, which consists of the cross-correlation of the received reflected signal with a clean replica of the accessible/public codes [12]. These codes used to have a narrower bandwidth than the encrypted codes. A more detailed description of both techniques can be found in [13]. Even though the iGNSS-R technique was developed earlier, in this section, the cGNSS-R signal model is presented prior to the iGNSS-R one because the iGNSS-R can be seen as the cGNSS-R with the addition of two extra noise terms.

### 2.1. cGNSS-R

The voltage signal after the correlation with a clean replica of the satellite code (waveform) has three main different components:

(1) $$y_{c}\left(t,\tau \right)=\frac{1}{T_{c}}\int_{-\frac{T_{c}}{2}}^{+\frac{T_{c}}{2}}\left(u_{r}\left(t+t^{\prime }+\tau \right)+n_{r_{t}}\left(t+t^{\prime }+\tau \right)\right)a\left(t+t^{\prime }\right)dt^{\prime }=\rho_{0}\left(\tau \right)+n_{S}\left(t,\tau \right)+n_{T,c}\left(t,\tau \right),$$

where *t* and *τ* stand for time and lag respectively, $u_{r}$ stands for the received reflected (*r*) signal, $n_{r_{t}}$ for the reflected thermal noise signal, *a* for the satellite spreading code, $T_{c}$ for the coherent integration time, subscript *c* for cGNSS-R, $\rho_{0}\left(\tau \right)$ is a deterministic value and stands for the coherent component of the signal, $n_{S}\left(t,\tau \right)$ is a complex Gaussian random variable with zero mean and power/variance $2\sigma_{s}^{2}\left(\tau \right)$ representing the incoherent reflected power or speckle noise [2,14], and $n_{T,c}\left(t,\tau \right)$ is also a complex Gaussian random variable with zero mean and power/variance $2\sigma_{t,c}^{2}\left(\tau \right)$, which represents the thermal noise in the cGNSS-R.

The signal part of the computed waveform can be expressed as a function of the system parameters as [15]:

(2) $$\begin{array}{cc} y_{s}\left(t,\tau \right) & =\frac{1}{T_{c}}\int_{-\frac{T_{c}}{2}}^{+\frac{T_{c}}{2}}u_{r}\left(t+t^{\prime }+\tau \right)a\left(t+t^{\prime }\right)dt^{\prime }=\int \sqrt{EIRP_{T}}D\left(\vec{\rho }\right)ACF\left[\delta t\left(t,\vec{\rho }\right)\right]S\left[\delta f\left(t,\vec{\rho }\right)\right]g\left(\vec{\rho },t\right)d\vec{\rho }\\ & =\rho_{0}\left(\tau \right)+n_{S}\left(t,\tau \right), \end{array}$$

where $\sqrt{EIRP_{T}}$ is the square root of the Equivalent Isotropically Radiated Power (EIRP) of the transmitting satellite, $D(\vec{\rho })$ is the voltage antenna pattern projected on ground, ACF stands for the Auto-Correlation Function (ACF) shape of the GNSS signals [16], *S* is a *sinc* function expressing the Doppler behavior of the spreading function [17], $\vec{\rho }$ is a vector from the specular reflection position to the surface scattering point, and $g(\vec{\rho },t)$ is defined as a solution for the scattered field in the Kirchoff Approximation (KA) [15]:

(3) $$g\left(\vec{\rho },t\right)=-r\frac{\exp\left(j2\pi ft\right)}{j4\pi R_{0}R}\exp\left[-j\kappa (R_{0}+R)\right]\frac{q^{2}}{q_{z}},$$

where *r* stands for the Fresnel reflection coefficient, *f* is the GNSS carrier frequency, *κ* stands for the wavenumber, $R_{0}$ is the distance from some surface point to the transmitter, *R* is the distance from the same surface point to the receiver, $\vec{q}=-\kappa \left(\vec{n}-\vec{m}\right)=\left(q_{z},{\vec{q}}_{⊥}\right)$, $\vec{m}$ is the unitary vector of the incident wave, and $\vec{n}$ is the unitary vector of the scattered wave.

For a perfectly flat surface, a purely coherent scattering reflection takes place. Then, Equation (2) tends to [15,18]

(4) $$y_{s}\left(t,\tau \right)∼-\sqrt{EIRP_{T}}D\left(0\right)ACF\left[\tau \right]S\left[0\right]r\frac{\exp\left[j2\pi ft-j2\pi \left(\frac{R_{0,sp}+R_{sp}}{\lambda }\right)\right]}{j4\pi (R_{0,sp}+R_{sp})}\exp\left[-2\kappa^{2}\sigma_{h}^{2}\cos^{2}\left(\theta_{inc}\right)\right],$$

where $\sigma_{h}^{2}$ stands for the variance of surface heights, $\theta_{inc}$ for the incidence angle, and the factor $\exp\left[-2\kappa^{2}\sigma_{h}^{2}\cos^{2}\left(\theta_{inc}\right)\right]$ corresponds to the reflected field attenuation due to surface roughness under the Physical Optics (PO) approximation [19]. Equation (4) is equivalent to the direct transmission case, an image of the receiver is located under the surface, and the incident field is multiplied by the Fresnel reflection coefficient [15]. This is in agreement with [18], where the specular reflected power in a bistatic configuration is defined. However, for very rough surfaces ($\kappa \sigma_{h}\sin\left(\theta_{inc}\right)\gg 1$), the coherently reflected component vanishes. As a result, the received scattered field becomes normally distributed, and its expression is given in [15], which represents a so-called “speckle” or self-noise.

The thermal noise part of the reflected waveform is expressed by

(5) $$y_{r_{t}}\left(t,\tau \right)=\frac{1}{T_{c}}\int_{-\frac{T_{c}}{2}}^{+\frac{T_{c}}{2}}n_{r_{t}}\left(t+t^{\prime }+\tau \right)a\left(t+t^{\prime }\right)dt^{\prime }=n_{T,c}\left(t,\tau \right),$$

### 2.2. iGNSS-R

For this case, the complex voltage signal after the correlation with the direct signal has also three distinct components, which are similar to those in the cGNSS-R case, taking into account the addition of some terms to the interferometric thermal noise component. Therefore, the iGNSS-R voltage waveform is

(6) $$y_{i}\left(t,\tau \right)=\frac{1}{T_{c}}\int_{-\frac{T_{c}}{2}}^{+\frac{T_{c}}{2}}\left[u_{r}\left(t+t^{\prime }+\tau \right)+n_{r_{t}}\left(t+t^{\prime }+\tau \right)\right]s_{d}\left(t+t^{\prime }\right)dt^{\prime }=\rho_{0}\left(\tau \right)+n_{S}\left(t,\tau \right)+n_{T,i}\left(t,\tau \right),$$

where $s_{d}$ stands for the direct sampled signal, and $n_{T,i}\left(t,\tau \right)$ for the iGNSS-R equivalent thermal noise term. If the direct signal is under the Line Of Sight (LOS) conditions, it can be expressed as a sum of the coherent term, which represents the signal code, and the thermal noise component for the direct signal. Consequently, the iGNSS-R waveform can be expressed as a function of the cGNSS-R waveform as follows:

(7) $$y_{i}\left(t,\tau \right)=y_{c}\left(t,\tau \right)+\frac{1}{\sqrt{SNR_{d}}}\left[y_{u_{r},d_{t}}\left(t,\tau \right)+y_{r_{t},d_{t}}\left(t,\tau \right)\right],$$

where $y_{c}\left(t,\tau \right)$ is given by Equation (1), $1/\sqrt{SNR_{d}}$ is a normalizing factor that appears due to normalizing the spreading code direct signal part to unit energy, $SNR_{d}$ is the pre-correlation thermal SNR for the direct signal, $y_{u_{r},d_{t}}\left(t,\tau \right)$ is the correlation of the reflected signal component with the direct channel thermal noise, and $y_{r_{t},d_{t}}\left(t,\tau \right)$ is the correlation between the direct and reflected noise components.

Equivalent waveform models can be found in several references for both the cGNSS-R and the iGNSS-R cases [7,8,9,15,20,21,22,23]. However, they were all based on rough sea scenarios and considered the widely accepted sea-surface scattering model proposed in [15] which neglects the presence of the coherent component. The signal model presented here does not neglect this component, and therefore it is more general.

## 3. Correlation Peak Statistics in GNSS and Squaring Loss Paradox

**Figure 1:**
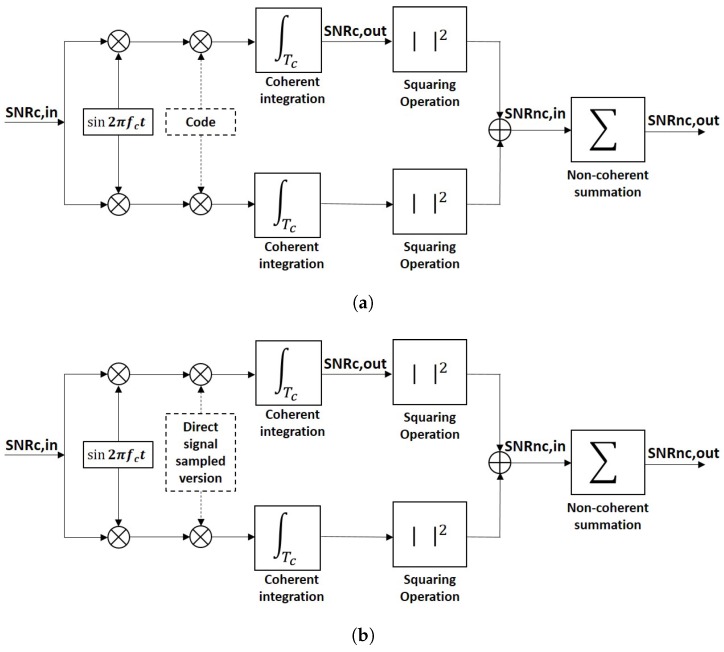
(**a**) Typical GNSS/cGNSS-R receiver block diagram; (**b**) Simplified iGNSS-R receiver block diagram.

The analysis of the correlation peak statistics has been widely explored in the GNSS literature [24,25,26,27,28]. The fundamental operation in a GNSS signal acquisition system is the cross-correlation of the digitized received signal with a clean replica of the satellite code (matched filter) in order to obtain the so-called waveform, see Equation (1), which is the same computational operation that is performed in a conventional GNSS-R receiver. This computation is also known as coherent integration, and it can last up to 20 ms, which is the duration of a navigation bit. Longer coherent integration times can be always applied after compensating for the navigation bit sign change. After coherent integration, non-coherent integration is performed, which consists of summing the waveforms obtained in power units to improve the visibility or detectability of the satellite presence. Non-coherent integration requires a squaring operation which changes the statistics of the obtained samples, and therefore, leads to a redefinition of the resulting SNR. Figure 1a shows a typical block diagram of a coherent or I/Q detector. It is possible to introduce four different definitions of the SNR, but they correspond, in fact, to two. The first one is the ${\mathrm{SNR}}_{c,\mathrm{in}}$, which is the SNR before correlation with the clean replica of the satellite code, or pre-correlation SNR. It is always negative since GNSS signals are below the noise level, unless a very high directivity antenna is used to acquire them. The second one is the ${\mathrm{SNR}}_{c,\mathrm{out}}$, which is the SNR after correlation with the clean replica of the satellite code. It is related to the ${\mathrm{SNR}}_{c,\mathrm{in}}$ by the signal’s bandwidth times the coherent integration time. Basically, the thermal noise bandwidth is reduced in the coherent integration process, letting the signal rise above the new thermal noise level. The third one is the ${\mathrm{SNR}}_{\mathrm{nc},\mathrm{in}}$, which is the SNR resulting from a non-coherent detection scheme where no phase information is available. The ${\mathrm{SNR}}_{\mathrm{nc},\mathrm{in}}$ is related to the ${\mathrm{SNR}}_{c,\mathrm{out}}$ by the squaring-loss parameter [29]. The last one is the ${\mathrm{SNR}}_{\mathrm{nc},\mathrm{out}}$, which is the SNR after the non-coherent integration/averaging.

In traditional GNSS applications, the received signal is formed by a coherent or LOS component and the thermal noise. The coherent component is a deterministic signal, and the thermal noise is modeled as a complex Gaussian random variable with zero mean and variance/power $2{\sigma_{T}}^{2}$. In this case, the incoherent scattered component term is not present since there is no scattering process involved. To analyze the ${\mathrm{SNR}}_{c,\mathrm{out}}$, it is necessary to concentrate on the correlation peak, where the signal’s amplitude is the largest. One way to determine the ${\mathrm{SNR}}_{c,\mathrm{out}}$ is by applying the detectability criterion [30], which is based on a comparison of the signal power against its variability (noise power)

(8) $$d=\frac{E\left\{f_{S+N}\right\}-E\left\{f_{N}\right\}}{\sqrt{E\left\{{f_{N}}^{2}\right\}-{E\{f_{N}\}}^{2}}},$$

where $f_{S+N}$ is a function with a subscript which stands for the signal (*S*) plus noise (*N*) components, and $f_{N}$ is a function with a subscript which indicates that stands only for the noise components.

The detectability criterion involves the use of the signal’s mean value which is divided it by its standard deviation. It is an amplitude/voltage signal-to-noise ratio if the function $f_{S+N}$ is defined in Volts [V] units, and it is a power signal-to-noise ratio if the same function is defined in Volts squared [V${}^{2}$] units [31]. If it is applied when the samples are squared, which is the Volts squared case, the conventional SNR definition is obtained as:

(9) $$d=\frac{E\left\{f_{S+N}\right\}-E\left\{f_{N}\right\}}{\sqrt{E\left\{{f_{N}}^{2}\right\}-{E\{f_{N}\}}^{2}}}=\frac{A^{2}}{2{\sigma_{T}}^{2}}=\frac{P_{S}}{P_{N}},$$

where *A* is the signal amplitude in [V], $P_{S}=A^{2}$ stands for the signal (*S*) power, and $P_{N}=2{\sigma_{T}}^{2}$ for the thermal noise (*N*) power.

As seen, in the computation of the traditional detectability criterion (*d*), the signal power is measured at the waveform’s correlation peak (where both signal and noise components are present, S+N). The noise power is evaluated outside the waveform correlation peak (where the signal is not present, *N*). However, in the squaring process, the properties of the signal change. Before squaring, the signal’s variability at the correlation peak and at any other correlation lag is the same, since only zero-mean thermal noise is present. Conversely, after squaring, outside the correlation peak an exponential random variable is found (square of a complex Gaussian random variable), whereas at the correlation peak, a non-central $\chi^{2}$ random variable emerges due to the presence of the LOS signal component. This means that the variability at lags outside the interval occupied by the main part of the ACF function is different from the one at the correlation peak. Consequently, the effect of the same noise is different depending on the correlation lag where it is analyzed. Therefore, its effect on the correlation peak cannot be studied when there is no signal presence, which could be done before squaring. At this point, the so-called “squaring-loss" parameter plays a role in the observed SNR, and a different detectability criterion ($d^{\prime }$) must be used to take into account the noise effect at the correlation peak [32], which is

(10) $$d^{\prime }=\frac{E\left\{f_{S+N}\right\}-E\left\{f_{N}\right\}}{\sqrt{E\left\{{f_{S+N}}^{2}\right\}-{E\{f_{S+N}\}}^{2}}}.$$

Comparing Equations (10) to (8), it can be seen that whereas the numerator remains the same, the denominator changes. The computation of $d^{\prime }$ involves computing the correlation peak variability under the presence of both signal and noise terms, which is a more realistic approach. This detectability criterion can also be applied to estimate the ${\mathrm{SNR}}_{c,\mathrm{out}}$. In the particular example of Equation (9), the result would be the same than the one obtained using Equation (8), because before squaring the mean of the noise samples is zero, a fact that changes due to squaring. By specifying terms in Equation (10) for the squaring case, the ${\mathrm{SNR}}_{\mathrm{nc},\mathrm{in}}$ and the ${\mathrm{SNR}}_{\mathrm{nc},\mathrm{out}}$ can be expressed in terms of the ${\mathrm{SNR}}_{c,\mathrm{out}}$ [31,32,33]:

(11) $$d^{\prime }={\mathrm{SNR}}_{\mathrm{nc},\mathrm{in}}=\frac{{\mathrm{SNR}}_{c,\mathrm{out}}}{\sqrt{1+2{\mathrm{SNR}}_{c,\mathrm{out}}}},$$

(12) $$d^{\prime }={\mathrm{SNR}}_{\mathrm{nc},\mathrm{out}}=\sqrt{N_{eff}}\frac{{\mathrm{SNR}}_{c,\mathrm{out}}}{\sqrt{1+2{\mathrm{SNR}}_{c,\mathrm{out}}}},$$

where $\sqrt{N_{eff}}$ is the effective number of averaged samples [13,34]. If S+N samples are independent, which is a valid approximation here because the signal term is deterministic, the variability at the correlation peak is due to thermal noise whose samples are independent by definition, and therefore $N_{eff}$ tends to *N*, being *N* the number of independent samples averaged. Although these equations are valid for the navigation case, the GNSS-R case adds an extra feature: the speckle noise due to the scattering [2,14]. This means that the variability of the correlation peak is not only due to thermal noise, but also due to the scattering process, and previous equations should be modified accordingly.

## 4. Correlation Peak SNR in cGNSS-R and iGNSS-R

Due to the low-power and high-phase noise of the GNSS reflected signals both cGNSS-R and iGNSS-R approaches tend to use ∼1 ms of coherent integration time and then apply the non-coherent summations/averaging, which means that they work with the power waveforms instead of the complex-value voltage waveforms. The power waveform, $Y_{a}\left(t,\tau \right)$, is defined as the absolute-value squared of the voltage waveform:

(13) $$Y_{a}\left(t,\tau \right)={\left|y_{a}\left(t,\tau \right)\right|}^{2},$$

where *a* stands for *c* or *i*, in order to distinguish between the conventional and interferometric techniques. Hence, the detectability criteria become, where mathematical details are in Appendix A and Appendix C,

(14) $$d_{c}=\frac{P_{\mathrm{coh}}\left(\tau \right)+P_{\mathrm{incoh}}\left(\tau \right)}{P_{T_{c}}\left(\tau \right)},$$

(15) $$d_{c}^{\prime }=\frac{1}{\sqrt{{\left(1+\frac{1}{{\mathrm{SNR}}_{{\mathrm{TH}}_{c}}}\right)}^{2}-{\left(1-\frac{1}{{\mathrm{SNR}}_{\mathrm{SP}}}\right)}^{2}}},$$

(16) $$d_{i}=\frac{P_{\mathrm{coh}}\left(\tau \right)+P_{\mathrm{incoh}}\left(\tau \right)+P_{T_{c}}\left(\tau \right)\frac{SNR_{r}}{SNR_{d}}}{P_{T_{c}}\left(\tau \right)\left(1+\frac{1}{SNR_{d}}\right)}=\approx d_{c}\frac{1}{1+\frac{1}{SNR_{d}}},$$

(17) $$d_{i}^{\prime }=\frac{1+\frac{1}{d_{c}}\frac{SNR_{r}}{SNR_{d}}}{\sqrt{{\left(1+\frac{1}{{\mathrm{SNR}}_{{\mathrm{TH}}_{i}}}\right)}^{2}-{\left(1-\frac{1}{{\mathrm{SNR}}_{\mathrm{SP}}}\right)}^{2}}},$$

where $P_{\mathrm{coh}}={\left|\rho_{0}\left(\tau \right)\right|}^{2}$, and it stands for the coherent reflected power, $P_{\mathrm{incoh}}=E\{|n_{S}\left(t,\tau \right){|}^{2}\}=2\sigma_{s}^{2}\left(\tau \right)$, and it stands for the incoherent reflected power, $P_{T_{c}}=E\{|n_{T,c}\left(t,\tau \right){|}^{2}\}=2\sigma_{t,c}^{2}\left(\tau \right)$, and it stands for the cGNSS-R thermal noise power, $SNR_{d}$ is the pre-correlation SNR or ${\mathrm{SNR}}_{c,\mathrm{in}}$ for the direct signal, $SNR_{r}$ is the pre-correlation SNR or ${\mathrm{SNR}}_{c,\mathrm{in}}$ for the reflected signal, and:

(18) $${\mathrm{SNR}}_{{\mathrm{TH}}_{c}}=\frac{P_{\mathrm{coh}}\left(\tau \right)+P_{\mathrm{incoh}}\left(\tau \right)}{P_{T_{c}}\left(\tau \right)},$$

(19) $${\mathrm{SNR}}_{\mathrm{SP}}=\frac{P_{\mathrm{coh}}\left(\tau \right)+P_{\mathrm{incoh}}\left(\tau \right)}{P_{\mathrm{incoh}}\left(\tau \right)},$$

(20) $${\mathrm{SNR}}_{{\mathrm{TH}}_{i}}=\frac{P_{\mathrm{coh}}\left(\tau \right)+P_{\mathrm{incoh}}\left(\tau \right)}{P_{T_{i}}\left(\tau \right)}=\frac{P_{\mathrm{coh}}\left(\tau \right)+P_{\mathrm{incoh}}\left(\tau \right)}{P_{T_{c}}\left(\tau \right)\left(1+\frac{1}{SNR_{d}}\left(SNR_{r}+1\right)\right)}=\frac{{\mathrm{SNR}}_{{\mathrm{TH}}_{c}}}{1+\frac{1}{SNR_{d}}\left(SNR_{r}+1\right)},$$

where ${\mathrm{SNR}}_{{\mathrm{TH}}_{c}}$ is the post-correlation thermal SNR or ${\mathrm{SNR}}_{c,\mathrm{out}}$ for the reflected signal in the cGNSS-R case, ${\mathrm{SNR}}_{\mathrm{SP}}$ is the signal to speckle noise ratio, and ${\mathrm{SNR}}_{{\mathrm{TH}}_{i}}$ is the equivalent post-correlation thermal SNR or ${\mathrm{SNR}}_{c,\mathrm{out}}$ for the reflected signal in the iGNSS-R case.

The difference between taking into account the variability of the signal at its correlation peak or away from it is clearly seen by comparing $d_{c}$ with $d_{c}^{\prime }$, and $d_{i}$ with $d_{i}^{\prime }$. When the variability at the correlation peak is considered, the detectability criterion is degraded ($d_{c}>d_{c}^{\prime }$ and $d_{i}>d_{i}^{\prime }$). Also, the cGNSS-R and iGNSS-R approaches can be compared using the detectability criteria. The comparison between $d_{c}$ and $d_{i}$ shows that the detectability criterion for the iGNSS-R is a degraded version of the cGNSS-R one. This occurs because for the iGNSS-R approach the thermal noise rises in comparison with the cGNSS-R approach due to the two extra noise terms to be considered. However, if $SNR_{d}\gg 1$, then $d_{i}\to d_{c}$. The same occurs when considering the $d_{c}^{\prime }$ and $d_{i}^{\prime }$, since ${\mathrm{SNR}}_{{\mathrm{TH}}_{c}}>{\mathrm{SNR}}_{{\mathrm{TH}}_{i}}$. Equally, if $SNR_{d}\gg 1$, then ${\mathrm{SNR}}_{{\mathrm{TH}}_{i}}\to {\mathrm{SNR}}_{{\mathrm{TH}}_{c}}$, and $d_{i}^{\prime }\to d_{c}^{\prime }$. There is another aspect that is related to the definition of the detectability criterion, which is that the mean noise level value in the iGNSS-R case is not subtracted at the correlation peak by using the one computed at lags away from the correlation peak. The remaining term is $P_{T_{c}}\left(\tau \right)\frac{SNR_{r}}{SNR_{d}}$, but since $SNR_{r}\ll SNR_{d}$, this term can be neglected.

The coherent, incoherent, and thermal noise powers presented above can be computed as [18]

(21) $$P_{\mathrm{coh}}=E\left\{{\left|\rho_{0}\left(t,\tau \right)\right|}^{2}\right\}=\frac{E_{T}G_{R}D_{R}^{2}\left(0\right)\Lambda^{2}\left[0\right]{|S\left[0\right]|}^{2}\lambda^{2}}{{\left(4\pi \right)}^{2}{\left(R_{0,sp}+R_{sp}\right)}^{2}}{|r\left(\theta_{inc}\right)|}^{2}e^{-4\kappa^{2}\sigma_{h}^{2}\cos^{2}\left(\theta_{inc}\right)},$$

(22) $$P_{\mathrm{incoh}}\left(\tau \right)=E\left\{{\left|n_{S}\left(t,\tau \right)\right|}^{2}\right\}=2\sigma_{S}^{2}\left(\tau \right)=\frac{E_{T}G_{R}}{{\left(4\pi \right)}^{2}}I_{A_{illpq}}\left(\tau \right),$$

(23) $$I_{A_{illpq}}\left(\tau \right)=\lambda^{2}\int_{A_{ill}}\frac{\sigma_{pq}^{0}\left(\vec{\rho }\right)D_{T}^{2}\left(\vec{\rho }\right)D_{R}^{2}\left(\vec{\rho }\right)ACF^{2}\left[\tau ,\vec{\rho }\right]{\left|S\left[\tau ,\vec{\rho }\right]\right|}^{2}}{4\pi R_{0}^{2}\left(\vec{\rho }\right)R^{2}\left(\vec{\rho }\right)}d^{2}\rho ,$$

where $A_{ill}$ stands for the illuminated area, $\sigma_{pq}^{0}$ for the radar cross-section at the incident *p* polarization and reflected *q* polarization,

(24) $$P_{T_{c}}\left(\tau \right)=k\left[T_{ant}+T_{0}\cdot \left(F-1\right)\right]B_{coh}=\frac{kT_{Nr}}{T_{c}}=2\sigma_{t,c}^{2}\left(\tau \right),$$

where $B_{coh}=1/T_{c}$, *k* is the Boltzmann constant, $T_{ant}$ stands for the antenna temperature, $T_{0}=290$ K, *F* for the noise figure of the receiving chain, and $T_{Nr}$ for the receiver’s equivalent noise temperature. If it is assumed that thermal noise is white, the dependence on *τ* can be neglected and the thermal noise power is $2\sigma_{t,c}^{2}$.

The coherent component is defined only for |τ|≤1, which corresponds to the ACF function of the GNSS Pseudo-Random Noise (PRN) codes, and the radiation comes from the first Fresnel zone area. On the other side, the incoherent component exists for different values of *τ*, which will depend on the surface roughness conditions. Note that increasing the transmitted power or the antenna gain does not improve the signal-to-speckle noise ratio (${\mathrm{SNR}}_{\mathrm{SP}}$), because the speckle noise, or also Rayleigh fading, is a multiplicative/scattering noise (self-noise) [2]. Note that when the coherent component is negligible the ${\mathrm{SNR}}_{\mathrm{SP}}=1$, which occurs in a backscattering geometry such as in Synthetic Aperture Radar (SAR) systems [35]. Also, note that ${\mathrm{SNR}}_{\mathrm{SP}}=1$ for this case, because this SNR is defined as a power SNR. If the voltage signals are considered, the result of the ${\mathrm{SNR}}_{\mathrm{SP}}$ would be the well-known 5.56 dB for the speckle noise [35], which corresponds to the ratio between the mean and the standard deviation of a Rayleigh random variable.

## 5. Effect of Non-Coherent Summations in the Detectability Criteria

Due to the low power, and consequently low SNR, of GNSS reflected signals, averaging or non-coherent summations of consecutive waveforms is needed to improve the quality of the data retrieved and reduce the degradation from the speckle noise. This is also known as non-coherent integration, and it is also the same procedure performed in conventional GNSS receivers as it was remarked in the last step of the signal processing flow chart in Figure 1. Mathematically, non-coherent averaging of consecutive power waveforms is modeled as

(25) $$W\left(t,\tau \right)=\frac{1}{N}\sum_{n=1}^{N}Y_{na}\left(t,\tau \right),$$

where $Y_{na}\left(t,\tau \right)$ stands for the power waveform, *n* for the waveform index, and *N* is the number of waveforms used in the summation. When non-coherent integration is applied, the variability of the signal is highly reduced, which helps to detect the waveform. This operation should theoretically improve the SNR by a factor of $\sqrt{N}$, as stated in Equation (11), when samples are independent. However, some airborne experimental data have shown that the improvement factor is sometimes smaller than that in the lags where the signal term is present, see for instance [33,34]. This fact is generally related to the platform’s height and speed, which are the parameters that determine the surface correlation time, since for airborne and space-borne conditions the surface can be considered frozen during the coherent integration time [15]. The Van Cittert-Zernike theorem can be used to obtain a rough estimation of the surface correlation time, taking into account the wavelength, the platform’s speed and height, the shape of the illuminating signal, and the incidence angle [8,36]. If the estimated surface correlation time is larger than the coherent integration time, the speckle noise term will be correlated, and consequently samples will not be independent. Conversely, under spaceborne conditions, experimental data have shown that samples are practically uncorrelated [37], which occurs because the platform is relatively faster. In other words, for spaceborne applications, the surface correlation time is close to 1 ms (coherent integration time) due to the receiving satellite orbit that determines the platform’s speed, whereas for airborne applications, the surface correlation time depends highly on the platform’s height and speed, whose parameters depend more on the aircraft used.

Since experimental spaceborne waveforms seem to be uncorrelated [37], one may think that the averaging could be performed even using partially overlapped waveforms (waveforms obtained using some common signal samples), which could improve the final SNR by reducing the waveform’s variability. To do it, a more general mathematical expression of the non-coherent integration must be used:

(26) $$Z\left(t,\tau \right)=\frac{1}{T}\int_{0}^{T}Y_{a}\left(t+t^{\prime },\tau \right)dt^{\prime },$$

which is the averaging definition of a random process when T→∞, being *T* the non-coherent integration time.

**Figure 2:**
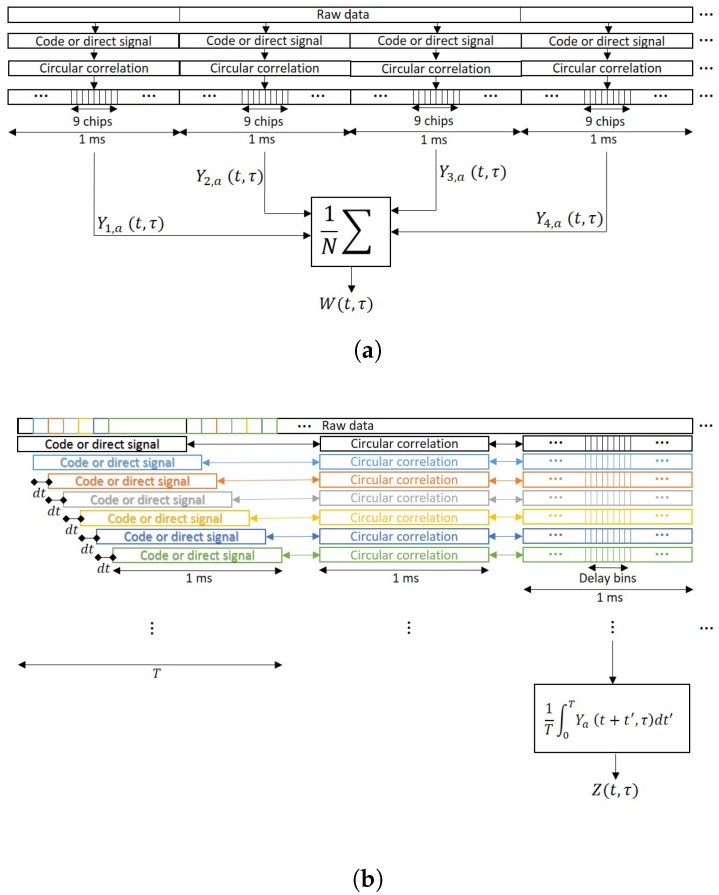
(**a**) Conventional non-coherent integration scheme; (**b**) General non-coherent integration definition.

The main differences between Equations (25) and (26) are depicted in Figure 2. While for Equation (25) the blocks of data for the incoherent averaging are taken separately without overlapping, for Equation (26) there is a moving window of 1 ms length and the waveforms are obtained using some overlapped samples. Note that in order to have all the waveforms aligned, i.e., to have the maximum correlation peak at the same lag, the clean C/A code block against whom the signal is correlated to must be circularly shifted. If 1 ms waveforms are highly correlated, the use of partially overlapped data data will not provide any improvement because the addition is made with data whose correlation coefficient is nearly 1.

Therefore, the detectability criteria after the non-coherent integration become, where the mathematical details can be found in Appendix D:

(27) $$d_{nc}=\sqrt{\frac{3}{2}\frac{T}{T_{coh}}}\frac{P_{\mathrm{coh}}\left(\tau \right)+P_{\mathrm{incoh}}\left(\tau \right)}{P_{T_{c}}\left(\tau \right)}=\sqrt{\frac{3}{2}\frac{T}{T_{coh}}}d_{c}$$

(28) $$d_{nc}^{\prime }=\frac{P_{\mathrm{coh}}\left(\tau \right)+P_{\mathrm{incoh}}\left(\tau \right)}{\sqrt{2{\bar{t}}_{s}P_{\mathrm{coh}}\left(\tau \right)P_{\mathrm{incoh}}\left(\tau \right)+2{\bar{t}}_{n}P_{\mathrm{coh}}\left(\tau \right)P_{T_{c}}\left(\tau \right)+2{\bar{t}}_{s}{\bar{t}}_{n}P_{\mathrm{incoh}}\left(\tau \right)P_{T_{c}}\left(\tau \right)+{\bar{T}}_{n}P_{T_{c}}^{2}\left(\tau \right)+{\bar{T}}_{s}P_{\mathrm{incoh}}^{2}\left(\tau \right)}}$$

(29) $$d_{ni}=\sqrt{\frac{3}{2}\frac{T}{T_{coh}}}\frac{P_{\mathrm{coh}}\left(\tau \right)+P_{\mathrm{incoh}}\left(\tau \right)+P_{T_{c}}\left(\tau \right)\frac{SNR_{r}}{SNR_{d}}}{\left(1+\frac{1}{SNR_{d}}\right)P_{T_{c}}\left(\tau \right)}=d_{nc}\frac{1}{1+\frac{1}{SNR_{d}}}+\sqrt{\frac{3}{2}\frac{T}{T_{coh}}}\frac{SNR_{r}}{SNR_{d}+1}$$

(30) $$d_{ni}^{\prime }=\frac{P_{\mathrm{coh}}\left(\tau \right)+P_{\mathrm{incoh}}\left(\tau \right)+P_{T_{c}}\left(\tau \right)\frac{SNR_{r}}{SNR_{d}}}{\sqrt{2{\bar{t}}_{s}P_{\mathrm{coh}}\left(\tau \right)P_{\mathrm{incoh}}\left(\tau \right)+2{\bar{t}}_{n}P_{\mathrm{coh}}\left(\tau \right)P_{T_{i}}\left(\tau \right)+2{\bar{t}}_{s}{\bar{t}}_{n}P_{\mathrm{incoh}}\left(\tau \right)P_{T_{i}}\left(\tau \right)+{\bar{T}}_{n}P_{T_{i}}^{2}\left(\tau \right)+{\bar{T}}_{s}P_{\mathrm{incoh}}^{2}\left(\tau \right)}}$$

where the normalized correlation times ${\bar{t}}_{s}$, ${\bar{t}}_{n}$, ${\bar{T}}_{s}$, and ${\bar{T}}_{n}$ are defined as:

(31a) $$\begin{array}{cc} {\bar{t}}_{s} & =\frac{1}{T}\int_{-T}^{T}\Lambda \left(\frac{\xi }{T}\right)\gamma_{s,s}\left(\xi ,\tau \right)d\xi , \end{array}$$

(31b) $$\begin{array}{cc} {\bar{t}}_{n} & =\frac{1}{T}\int_{-T}^{T}\Lambda \left(\frac{\xi }{T}\right)\gamma_{n_{T_{c}},n_{T_{c}}}\left(\xi ,\tau \right)d\xi =\frac{T_{c}}{T}, \end{array}$$

(31c) $$\begin{array}{cc} {\bar{T}}_{s} & =\frac{1}{T}\int_{-T}^{T}\Lambda \left(\frac{\xi }{T}\right){|\gamma_{s,s}\left(\xi ,\tau \right)|}^{2}d\xi , \end{array}$$

(31d) $$\begin{array}{cc} {\bar{T}}_{n} & =\frac{1}{T}\int_{-T}^{T}\Lambda \left(\frac{\xi }{T}\right){|\gamma_{n_{T_{c}},n_{T_{c}}}\left(\xi ,\tau \right)|}^{2}d\xi =\frac{2}{3}\frac{T_{c}}{T}. \end{array}$$

The normalized correlation times related to thermal noise (${\bar{t}}_{n}$, and ${\bar{T}}_{n}$) are $\frac{T_{c}}{T}$ and $\frac{2}{3}\frac{T_{c}}{T}$ respectively, and they show how effective is the incoherent averaging in thermal noise variability reduction. They are both equal in the cGNSS-R and iGNSS-R cases, as they depend on the normalized correlation function, which is equal in both cases; see Appendix A for the demonstration of equal thermal noise correlation functions. The same occurs with the incoherent power normalized correlation times (${\bar{t}}_{s}$, and ${\bar{T}}_{s}$), which strictly depend on the speed of the receiving platform, its height, and the spreading/modulation codes used, since the surface can be considered frozen during the coherent integration time. Note that for Equations (27), (29) and (31d) there is an improvement factor of $\sqrt{3/2}$ or 2/3, respectively. This occurs because the squaring operation performed to obtain the power waveform changes the correlation functions of the voltage waveforms, and what was a triangular correlation function now becomes a triangle squared. This factor 2/3 is the area of a normalized triangle squared function. Also note that this factor only helps in the reduction of the thermal noise variability, but not in the speckle noise, which depends on ${\bar{t}}_{s}$ and ${\bar{T}}_{s}$. However, if overlapped samples are not used, ${\bar{T}}_{n}$ becomes $\frac{T_{c}}{T}$. This last point would occur because the second and fourth order correlation functions of the thermal noise would become a Kronecker delta since when sampling a normalized triangle function or a normalized triangle squared function every ms the result is the Kronecker delta function. In such case, the factor $\sqrt{3/2}$ would disappear.

There is another aspect to highlight related to Equations (28) and (30). In those cases, there are several terms in the denominator, each of them related to different moments of the two relevant noises. If the thermal noise is the dominating noise term and its power is larger than the signal power considering both coherent and incoherent components, then the ${\bar{T}}_{n}$ is the dominating factor and the $\sqrt{3/2}$ gain will be seen here. Note, that if the thermal noise power is larger than the signal power the waveform shape is too noisy for any retrieval. If the thermal noise is the dominating noise term but the coherent signal power is larger than the thermal noise power, the SNR will be driven by the ${\bar{t}}_{n}$ parameter and no $\sqrt{3/2}$ improvement will be seen. However, if the speckle noise is the dominating noise term, two different things may occur. One is that the the coherent power is negligible and the SNR is driven by ${\bar{T}}_{s}$. The other one is that both coherent and incoherent signal powers are much larger than the thermal noise power, and therefore the SNR will be driven by the ${\bar{t}}_{s}$ parameter.

## 6. Correlation Peak Variability

The SNR has been defined with the help of the detectability criterion, which is basically the signal’s mean value divided by its standard deviation. Using those defined SNRs, the correlation peak variability should be computed in order to estimate the minimum incoherent integration time to obtain a peak variability lower than the accepted one, which indicates the system’s accuracy. The useful signal can be estimated as:

(32) $${\hat{S}}_{use}=S_{meas,S+N}-S_{meas,N}$$

where use stands for useful, and its standard deviation is:

(33) $$\sigma_{{\hat{S}}_{use}}=\sqrt{E\left\{{\hat{S}}_{use}^{2}\right\}-E{\left\{{\hat{S}}_{use}\right\}}^{2}}=\sqrt{\mathrm{Var}\left\{S_{meas,S+N}\right\}+\mathrm{Var}\left\{S_{meas,N}\right\}}$$

under the assumption that measurements of the signal + noise term (S+N) and the noise term (*N*) are uncorrelated, which is true since they are computed at different values of *τ* (different correlation lags), and the correlation functions derived in Appendix A demonstrate this point. Therefore, the variability of the signal for the cGNSS-R technique is (the $var\{S_{meas,S+N}\}$ and $var\{S_{meas,N}\}$ are computed in Appendix D):

(34) $$\sigma_{{\hat{S}}_{use},cGNSS-R}=\sqrt{2{\bar{t}}_{s}P_{\mathrm{coh}}\left(\tau \right)P_{\mathrm{incoh}}\left(\tau \right)+2{\bar{t}}_{n}P_{\mathrm{coh}}\left(\tau \right)P_{T_{c}}\left(\tau \right)+2{\bar{t}}_{s}{\bar{t}}_{n}P_{\mathrm{incoh}}\left(\tau \right)P_{T_{c}}\left(\tau \right)+2{\bar{T}}_{n}P_{T_{c}}^{2}\left(\tau \right)+{\bar{T}}_{s}P_{\mathrm{incoh}}^{2}\left(\tau \right)},$$

or normalized to the signal power ($P_{\mathrm{coh}}\left(\tau \right)+P_{\mathrm{incoh}}\left(\tau \right)$):

(35) $${\bar{\sigma }}_{{\hat{S}}_{use},cGNSS-R}=\sqrt{2\left(1-\frac{1}{{\mathrm{SNR}}_{\mathrm{SP}}}\right)\left(\frac{{\bar{t}}_{s}}{{\mathrm{SNR}}_{\mathrm{SP}}}+\frac{{\bar{t}}_{n}}{{\mathrm{SNR}}_{{\mathrm{TH}}_{c}}}\right)+2\frac{{\bar{t}}_{s}}{{\mathrm{SNR}}_{\mathrm{SP}}}\frac{{\bar{t}}_{n}}{{\mathrm{SNR}}_{{\mathrm{TH}}_{c}}}+2\frac{{\bar{T}}_{n}}{{\mathrm{SNR}}_{{\mathrm{TH}}_{c}}^{2}}+\frac{{\bar{T}}_{s}}{{\mathrm{SNR}}_{\mathrm{SP}}^{2}}}.$$

For the iGNSS-R technique such variability is:

(36) $$\sigma_{{\hat{S}}_{use},iGNSS-R}=\sqrt{2{\bar{t}}_{s}P_{\mathrm{coh}}\left(\tau \right)P_{\mathrm{incoh}}\left(\tau \right)+2{\bar{t}}_{n}P_{\mathrm{coh}}\left(\tau \right)P_{T_{i}}\left(\tau \right)+2{\bar{t}}_{s}{\bar{t}}_{n}P_{\mathrm{incoh}}\left(\tau \right)P_{T_{i}}\left(\tau \right)+\left(2-\frac{SNR_{r}}{SNR_{d}}\right){\bar{T}}_{n}P_{T_{i}}^{2}\left(\tau \right)+{\bar{T}}_{s}P_{\mathrm{incoh}}^{2}\left(\tau \right)},$$

which for the case $SNR_{r}\ll SNR_{d}$ can be approximated by:

(37) $$\sigma_{{\hat{S}}_{use},iGNSS-R}\approx \sqrt{2{\bar{t}}_{s}P_{\mathrm{coh}}\left(\tau \right)P_{\mathrm{incoh}}\left(\tau \right)+2{\bar{t}}_{n}P_{\mathrm{coh}}\left(\tau \right)P_{T_{i}}\left(\tau \right)+2{\bar{t}}_{s}{\bar{t}}_{n}P_{\mathrm{incoh}}\left(\tau \right)P_{T_{i}}\left(\tau \right)+2{\bar{T}}_{n}P_{T_{i}}^{2}\left(\tau \right)+{\bar{T}}_{s}P_{\mathrm{incoh}}^{2}\left(\tau \right)}.$$

If it is normalized by the signal power, it turns into:

(38) $${\bar{\sigma }}_{{\hat{S}}_{use},iGNSS-R}\approx \sqrt{2\left(1-\frac{1}{{\mathrm{SNR}}_{\mathrm{SP}}}\right)\left(\frac{{\bar{t}}_{s}}{{\mathrm{SNR}}_{\mathrm{SP}}}+\frac{{\bar{t}}_{n}}{{\mathrm{SNR}}_{{\mathrm{TH}}_{i}}}\right)+2\frac{{\bar{t}}_{s}}{{\mathrm{SNR}}_{\mathrm{SP}}}\frac{{\bar{t}}_{n}}{{\mathrm{SNR}}_{{\mathrm{TH}}_{i}}}+2\frac{{\bar{T}}_{n}}{{\mathrm{SNR}}_{{\mathrm{TH}}_{i}}^{2}}+\frac{{\bar{T}}_{s}}{{\mathrm{SNR}}_{\mathrm{SP}}^{2}}}.$$

## 7. Estimation of the SNR and Signal’s Peak Variability for the UK TDS-1 and GEROS-ISS Missions

In this section the derived theoretical values are applied to specific scenarios considering the lag (*τ*) where the signal is maximum (peak value) in order to estimate both the best achievable SNR and the variability of the measured reflectivity or radar cross section at that point. With those estimations, the scatterometric accuracy of the cGNSS-R and iGNSS-R techniques could be assessed. For the first scenario, it is considered the UK TDS-1 mission, launched in 2014 with a Global Positioning System (GPS) bistatic payload. For the second scenario, the future GEROS-ISS mission is considered, which is analyzed for both the cGNSS-R and iGNSS-R cases. For all these scenarios, it is assumed that only incoherent sea surface scattering takes place, so the coherent component is negligible. In such situations a widely accepted scattering model to simulate the incoherent reflected signal power is used [15], in which the radar cross-section is given by:

(39) $$\sigma_{0}\left(\vec{\rho }\right)=\frac{{\pi |r|}^{2}q^{4}}{q_{z}^{4}}P\left(-\frac{{\vec{q}}_{⊥}}{q_{z}}\right).$$

The theoretical results presented in this work have been extended to situations when there is a coherent component, and in those situations a different radar cross section model must be used. Also they have been extended to other lags using the appropriate correlation functions derived in the Appendices.

### 7.1. cGNSS-R

#### 7.1.1. UK TDS-1 Scenario

The main parameters of the simulation are shown in Table 1. For this scenario two different values of the received power at the Earth surface are considered, from which the EIRP of the GPS satellites is estimated. One is −158.5 dBW which is the minimum received power at the Earth’s surface defined by the GPS Interface Control Document (ICD) [38], and the other one is −153 dBW, which is the maximum received power at the Earth’s surface specified in the same document [38]. Both can be considered as pessimistic and optimistic cases, respectively. Also, all simulation results shown here consider 1 ms coherent integration time.

**Figure 3:**
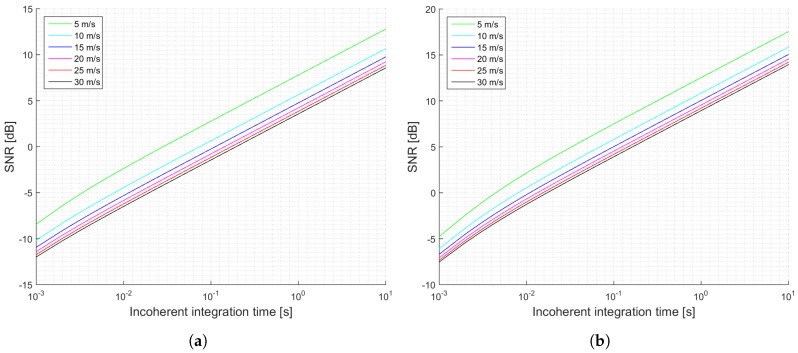
Simulations SNR for the TDS-1 scenario and cGNSS-R: (**a**) Minimum received power on ground of −158.5 dBW; (**b**) Minimum received power on ground of −153 dBW. Legend indicates u${}_{10}$ wind speed.

**Figure 4:**
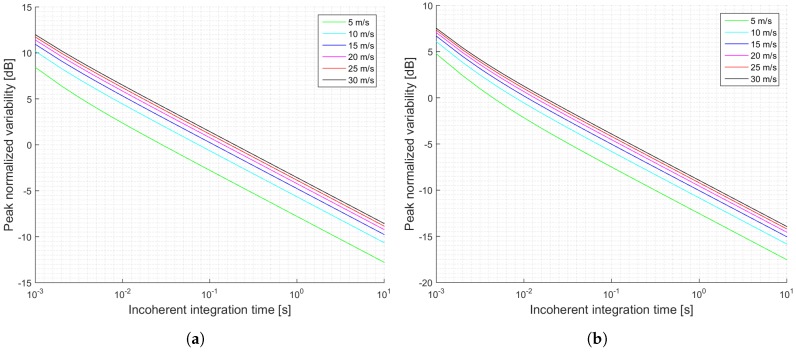
Simulations of the normalized peak variability for the TDS-1 scenario and cGNSS-R: (**a**) Minimum received power on ground of −158.5 dBW; (**b**) Minimum received power on ground of −153 dBW. Legend indicates u${}_{10}$ wind speed.

Figure 3 shows a summary of the estimated SNRs as a function of the incoherent integration time for the two proposed scenarios: a pessimistic one (a), and an optimistic one (b). This examples, which are truncated to 1 s of incoherent averaging, could be referred to the level 1b of the data provided by UK TDS-1 Measurement of Earth Reflected Radio-navigation Signals By Satellite (MERRByS) research team. Figure 4 shows the estimated normalized peak variability or accuracy of the $\sigma_{0}$ retrieval for the scenario described in Table 1 and the estimated SNRs shown in Figure 3.

#### 7.1.2. GEROS-ISS Scenario

The main parameters of the simulation are shown in Table 2. For this scenario the same parameters as for the UK TDS-1 scenario have been considered only changing the receiving antenna directivity, and the platform’s height and speed, which will change the ${\bar{t}}_{s}$ and ${\bar{T}}_{s}$ parameters. Also, all simulation results shown here use 1 ms coherent integration time.

**Figure 5:**
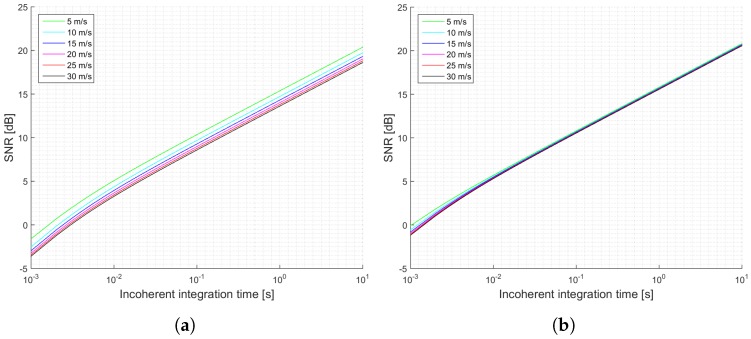
Simulations SNR for the GEROS-ISS scenario and cGNSS-R: (**a**) Minimum received power on ground of −158.5 dBW; (**b**) Minimum received power on ground of −153 dBW. Legend indicates u${}_{10}$ wind speed.

**Figure 6:**
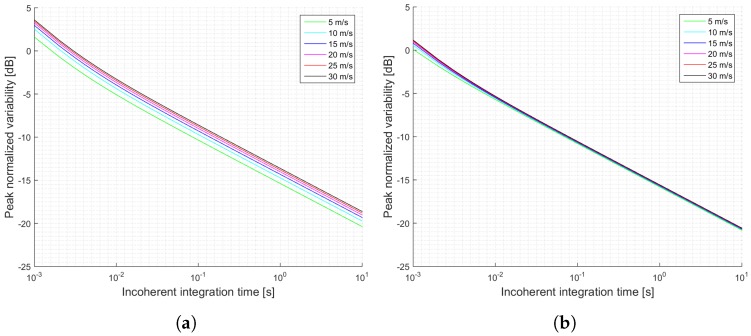
Simulations of the normalized peak variability for the GEROS-ISS scenario and cGNSS-R: (**a**) Minimum received power on ground of −158.5 dBW; (**b**) Minimum received power on ground of −153 dBW. Legend indicates u${}_{10}$ wind speed.

Figure 5 shows another summary of the estimated SNRs for the GEROS-ISS mission as a function of the incoherent integration time for the two different values of received power. These simulations can be used to determine the expected SNR and better define the parameters of the cGNSS-R scatterometric operation mode. Figure 6 shows the estimated normalized peak variability for the estimated SNRs shown in Figure 5. It is seen that for the most optimistic case, the expected performance does not depend on the sea state, because it is largely determined by the speckle noise.

### 7.2. iGNSS-R

#### GEROS-ISS Scenario

**Figure 7:**
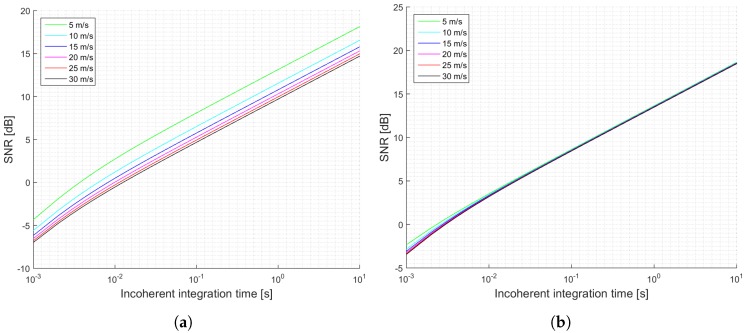
Simulations SNR for the GEROS-ISS scenario and iGNSS-R: (**a**) Total EIRP of 28.64 dBW (pessimistic); (**b**) Total EIRP of 34.23 dBW (optimistic). Legend indicates u${}_{10}$ wind speed.

**Figure 8:**
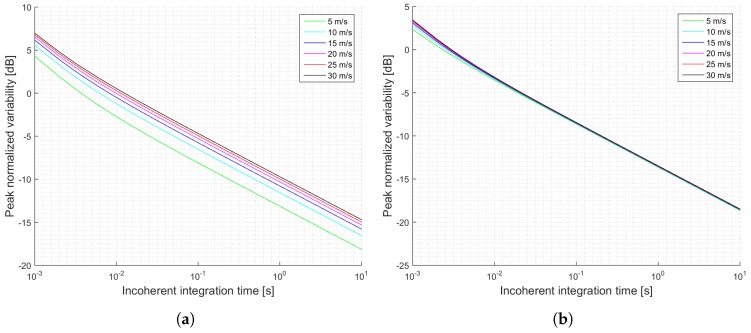
Simulations of the normalized peak variability for the GEROS-ISS scenario and iGNSS-R: (**a**) Total EIRP of 28.64 dBW (pessimistic); (**b**) EIRP of 34.23 dBW (optimistic). Legend indicates u${}_{10}$ wind speed.

The main parameters of the simulation are shown in Table 3 and Table 4 for the pessimistic and optimistic cases, respectively. For the iGNSS-R technique the same parameters as for the GEROS-ISS scenario have been considered while only changing the traditional waveform (C/A code) to the full-composite model (C/A- , P-, and M-codes), which will change the ${\bar{t}}_{s}$ and ${\bar{T}}_{s}$ parameters. Note that each signal term for each code will result in a different correlation time (the chip size determines the footprint size at the reflecting surface), and the correlation function can be expressed as a weighted linear combination of each code correlation function. Consequently, the EIRPs have been changed and separated by the code under use, to finally add them up and obtain the total EIRP. Furthermore, in the iGNSS-R the bandwidth is a critical parameter, since it determines the $SNR_{d}$ and $SNR_{r}$, which at the same time determines the scatterometric accuracy. They do not depend on the coherent integration time because they refer to the pre-correlation SNR. Results for this simulations are shown in Figure 7 for the SNR, and in Figure 8 for the normalized peak variability.

## 8. Discussion

The previous section has shown several simulations for different scenarios. Firstly, it can be concluded that when the antenna directivity is relatively low, which is the case of the UK TDS-1 scenario, the signal power is an important parameter, since it increases the SNR, and decreases the signal’s variability. This effect can be observed by comparing Figure 3a,b and Figure 4a,b. This indicates that for those scenarios the thermal SNR is the limiting factor. The change in the slope in those scenarios is justified because first it is dominating the term that multiplies ${\bar{T}}_{n}$ and after several averages the term that dominates is the one that multiplies ${\bar{t}}_{n}$.

When the antenna directivity is large enough, which is in the case of the proposed antenna for the GEROS-ISS mission, the transmitted power is not that important, and the expected performance does not depend significantly on the wind speed. This can be seen by comparing Figure 5a,b and Figure 6a,b. Furthermore, an increase on the transmitted power by the GPS satellites results in a retrieval performance independent from the wind speed. When the antenna has a directivity of 23 dB, the incoherent power is one order of magnitude larger than the thermal noise power, and therefore it is the factor determining the SNR.

When comparing the cGNSS-R and the iGNSS-R techniques, the results of the expected SNR and the peak variability are at least 3 dB better for the cGNSS-R considering the same simulation conditions. This occurs mainly because the thermal SNR for the iGNSS-R is degraded as compared to the cGNSS-R one. Also, there is another aspect to be analyzed: the wider bandwidth codes used in the iGNSS-R translate into smaller footprints, resulting in a larger correlation time between waveforms, and a reduction of the improvement by incoherent averaging is expected as compared to the cGNSS-R approach. This is seen in the slope of the SNR graphs. For cGNSS-R approach it is a little bit larger than for the iGNSS-R. However, due to the high speed of the spaceborne platform, for the simulation conditions they were very similar.

Note that the incoherent averaging considered here includes partially overlapped waveforms, and in the case when they are not partially overlapped, the simulations presented are an overestimation of the expected performance. In that situation, ${\bar{T}}_{n}$ would increase, resulting in a degradation of the expected SNR and an increase of the peak’s variability (the factor $\sqrt{3/2}$ would become 1). However, if the antenna directivity is as large as the one in the GEROS-ISS mission, the limiting factor is the speckle noise rather than the thermal noise, and experimental results will be closer to the theoretical ones.

All equations derived in this work can be applied to any lag different from the specular one, taking into account that the coherent component in those cases will be negligible. All the necessary correlation functions are available in the Appendices, and the correlation times should be recomputed accordingly for the appropriate lag. If the surface region under analysis falls into the delay-Doppler ambiguity free zone, the Van Cittert-Zernike theorem can be used to compute the correlation times. However, if delay-Doppler ambiguity exists, it should be computed taking into account two different areas contributing to the same delay-Doppler cell.

## 9. Conclusions

This work has analyzed the expected SNR and estimated $\sigma_{0}$ variability for cGNSS-R and iGNSS-R from a theoretical point of view including the presence of a coherent scattering component, which had been neglected in previous works. Recent UK TDS-1 data shows that for some types of surfaces the coherent component cannot be disregarded, and therefore the reflected signal does not always obey Gaussian statistics, but a Hoyt one.

Theoretical expressions of the expected SNR and the estimated $\sigma_{0}$ variability are presented in this work which allow to predict the scatterometric performance of any GNSS-R mission. The first important point is that, if the antenna directivity is not large enough, thermal SNR is the main limiting factor of the scatterometric performance. However, if it is sufficiently large, speckle noise becomes the limiting factor, and in that case, the cGNSS-R always performs better than the iGNSS-R because the equivalent thermal noise is lower. The second important point is that, the larger the transmitted power or the larger the directivity, the lower the dependence on the wind speed. The directivity threshold when the speckle noise is the entirely dominant term lies on 23 dB.

The averaging of partially overlapped waveforms has also been proposed and analyzed in this work, as it would help to reduce the signal variability induced by thermal noise up to 0.88 dB. However, this technique is mainly applicable to spaceborne scenarios where the platform moves faster enough, and the surface correlation time is around 1–2 ms, while under airborne situations the surface correlation time may be sometimes larger and speckle noise would become the limiting factor.

## Figures and Tables

**Table 1:** UK TDS-1 scenario simulation parameters.

Sensor Parameter	Magnitude
Orbit Height	635 [km]
Ground speed	6864 [m/s]
Minimum Rx Power on Earth	−158.5 [dBW]
Maximum Rx Power on Earth	−153 [dBW]
Incidence angle	15°
Frequency Band	L1 (C/A Code)
Sea Water Dielectric Constant	72.6 + j58.5
Down-Looking Antenna Gain	13 [dBiC]
Noise Figure	3.5 [dB]

**Table 2:** GEROS-ISS scenario simulation parameters.

Sensor Parameter	Magnitude
Orbit Height	400 [km]
Ground speed	7214 [m/s]
Minimum Rx Power on Earth	−158.5 [dBW]
Maximum Rx Power on Earth	−153 [dBW]
Incidence angle	15°
Frequency Band	L1 (C/A Code)
Sea Water Dielectric Constant	72.6 + j58.5
Down-Looking Antenna Gain	22 [dBiC]
Noise Figure	3.5 [dB]

**Table 3:** GEROS-ISS scenario simulation parameters for the iGNSS-R pessimistic case.

Sensor Parameter	Magnitude
EIRP C/A	24 [dBW]
EIRP M	25.5 [dBW]
EIRP P	21 [dBW]
EIRP Total	28.64 [dBW]
Orbit Height	400 [km]
Ground speed	7214 [m/s]
Incidence angle	15°
Frequency Band	L1 (Composite)
Sea Water Dielectric Constant	72.6 + j58.5
Up-Looking Antenna Gain	22 [dBiC]
Down-Looking Antenna Gain	22 [dBiC]
Noise Figure	3.5 [dB]
Bandwidth	40 [MHz]

**Table 4:** GEROS-ISS scenario simulation parameters changes for the iGNSS-R optimistic case.

Sensor Parameter	Magnitude
EIRP C/A	29.5 [dBW]
EIRP M	31 [dBW]
EIRP P	27 [dBW]
EIRP Total	34.23 [dBW]

## Abbreviations

The following abbreviations are used in this manuscript:

ACF	Auto-Correlation Function
cGNSS-R	conventional GNSS-R
DDM	Delay-Doppler Map
EIRP	Equivalent Isotropically Radiated Power
GEROS-ISS	GNSS REflectometry, Radio Occultation and Scatterometry on board the ISS
GNSS	Global Navigation Satellite Systems
GNSS-R	GNSS-Reflectometry
GPS	Global Positioning System
ICD	Interface Control Document
iGNSS-R	interferometric GNSS-R
ISS	International Space Station
KA	Kirchoff Approximation
LOS	Line Of Sight
MERRByS	Measurement of Earth Reflected Radio-navigation Signals By Satellite
PARIS	PAssive Reflectometry and Interferometry System
PO	Physical Optics
PRN	Pseudo-Random Noise
RF	Radio Frequency
SNR	Signal-to-Noise Ratio
UK	United Kingdom
UK TDS-1	UK TechDemoSat-1

## Appendix A. Correlation Functions of the Different Terms

This Appendix details the computation of the correlation function of the different terms of the waveform for both the cGNSS-R and iGNSS-R cases. This is used to estimate each component power contribution and in the other Appendices where higher order statistics must be computed. This Appendix includes the general signal model presented in this work, and the one used to apply the signal processing algorithm described in Figure 2b (overlapped waveforms).

The correlation function of $n_{T,c}\left(t,\tau \right)$ is:

(A1) $$\begin{array}{cc} E\{n_{T,c}\left(t_{1},\tau_{1}\right)n_{T,c}^{*}\left(t_{2},\tau_{2}\right)\}= & \frac{1}{T_{c}^{2}}E\left\{\int_{-\frac{T_{c}}{2}}^{+\frac{T_{c}}{2}}n_{r_{t}}\left(t_{1}+t^{\prime }+\tau_{1}\right)a\left(t_{1}+t^{\prime }\right)dt^{\prime }\int_{-\frac{T_{c}}{2}}^{+\frac{T_{c}}{2}}n_{r_{t}}^{*}\left(t_{2}+t^{\prime \prime }+\tau_{2}\right)a\left(t_{2}+t^{\prime \prime }\right)dt^{\prime \prime }\right\}=\\ = & \frac{1}{T_{c}^{2}}\int_{-\frac{T_{c}}{2}}^{+\frac{T_{c}}{2}}dt^{\prime }\int_{-\frac{T_{c}}{2}}^{+\frac{T_{c}}{2}}dt^{\prime \prime }a\left(t_{1}+t^{\prime }\right)a\left(t_{2}+t^{\prime \prime }\right)E\left\{n_{r_{t}}\left(t_{1}+t^{\prime }+\tau_{1}\right)n_{r_{t}}^{*}\left(t_{2}+t^{\prime \prime }+\tau_{2}\right)\right\}. \end{array}$$

Note that $t_{1}$ and $t_{2}$ stand for different times, $\tau_{1}$ and $\tau_{2}$ for different delays/lags. The term $E\{n_{r_{t}}\left(t_{1}+t^{\prime }+\tau_{1}\right)n_{r_{t}}^{*}\left(t_{2}+t^{\prime \prime }+\tau_{2}\right)\}$ stands for the correlation function of the incident thermal noise, which is $R_{n_{r_{t}},n_{r_{t}}}\left(t_{1}-t_{2}+\tau_{1}-\tau_{2}+t^{\prime }-t^{\prime \prime }\right)$, and it can be obtained assuming a band-limited white noise spectrum (square pulse in the frequency domain) and computing its inverse Fourier transform. Therefore:

(A2) $$E\left\{n_{T,c}\left(t_{1},\tau_{1}\right)n_{T,c}^{*}\left(t_{2},\tau_{2}\right)\right\}=\frac{1}{T_{c}^{2}}\int_{-\frac{T_{c}}{2}}^{+\frac{T_{c}}{2}}dt^{\prime }\int_{-\frac{T_{c}}{2}}^{+\frac{T_{c}}{2}}dt^{\prime \prime }a\left(t_{1}+t^{\prime }\right)a\left(t_{2}+t^{\prime \prime }\right)kT_{Nr}\frac{\sin\left(\pi B(t_{1}-t_{2}+\tau_{1}-\tau_{2}+t^{\prime }-t^{\prime \prime })\right)}{\pi (t_{1}-t_{2}+\tau_{1}-\tau_{2}+t^{\prime }-t^{\prime \prime })},$$

where *B* is the Radio Frequency (RF) bandwidth of the system, and $kT_{Nr}$ the reflected thermal noise spectral density. Also, assuming that $1/B\ll T_{c}$ the term $\frac{\sin\left(\pi B(t_{1}-t_{2}+\tau_{1}-\tau_{2}+t^{\prime }-t^{\prime \prime })\right)}{\pi (t_{1}-t_{2}+\tau_{1}-\tau_{2}+t^{\prime }-t^{\prime \prime })}$ can be approximated by a *δ* function in the integral. Therefore:

(A3) $$\begin{array}{cc} E\{n_{T,c}\left(t_{1},\tau_{1}\right)n_{T,c}^{*}\left(t_{2},\tau_{2}\right)\}= & \frac{kT_{Nr}}{T_{c}^{2}}\int_{-\frac{T_{c}}{2}}^{+\frac{T_{c}}{2}}dt^{\prime }\int_{-\infty }^{+\infty }dt^{\prime \prime }a\left(t_{1}+t^{\prime }\right)a\left(t_{2}+t^{\prime \prime }\right)\Pi \left(\frac{t^{\prime \prime }-T_{c}/2}{T_{c}}\right)\times \\ & \delta (t_{1}-t_{2}+\tau_{1}-\tau_{2}+t^{\prime }-t^{\prime \prime })=\\ = & \frac{kT_{Nr}}{T_{c}^{2}}\int_{-\frac{T_{c}}{2}}^{+\frac{T_{c}}{2}}a\left(t_{1}+t^{\prime }\right)a\left(t_{1}+t^{\prime }+\tau_{1}-\tau_{2}\right)\Pi \left(\frac{t_{1}+t^{\prime }+\tau_{1}-\tau_{2}-t_{2}-T_{c}/2}{T_{c}}\right)dt^{\prime }, \end{array}$$

and if this correlation is analyzed for the same delay ($\tau_{1}=\tau_{2}$), then:

(A4) $$E\left\{n_{T,c}\left(t_{1},\tau \right)n_{T,c}^{*}\left(t_{2},\tau \right)\right\}=\frac{kT_{Nr}}{T_{c}}\Lambda \left(\frac{t_{1}-t_{2}}{T_{c}}\right)=2\sigma_{t,c}^{2}\left(\tau \right)\gamma_{n_{Tc},n_{Tc}}\left(t_{1}-t_{2},\tau =0\right),$$

where $\gamma_{n_{Tc},n_{Tc}}\left(t_{1}-t_{2},\tau =0\right)$ is the normalized correlation function of the thermal noise after the correlation with a clean replica of the satellite code, and Λ refers to the triangle function which is defined as:

(A5) $$\Lambda \left(\frac{\xi }{T}\right)=\left\{\begin{array}{cc} 1-\frac{\left|\xi \right|}{T} & |\xi |\leq T\\ 0 & elsewhere \end{array}\right..$$

The general expression of the correlation function becomes:

(A6) $$E\left\{n_{Tc}\left(t_{1},\tau_{1}\right)n_{Tc}^{*}\left(t_{2},\tau_{2}\right)\right\}=\frac{kT_{Nr}}{T_{c}}\Lambda \left(\frac{t_{1}-t_{2}}{T_{c}}\right)R_{a,a}\left(\tau_{1}-\tau_{2}\right),$$

where the $R_{a,a}\left(\tau_{1}-\tau_{2}\right)=\Lambda \left(\frac{\tau_{1}-\tau_{2}}{\tau_{chip}}\right)$, and it is the code auto-correlation function [16] which is approximated by the Λ function in the GPS case, where $\tau_{chip}$ is the chip length (977 ns for the GPS C/A code). Note that white thermal noise is by definition uncorrelated, but due to the coherent integration process it becomes partially correlated for the same delay *τ*. Also note that by the properties of the Fourier transform, when $t_{1}=t_{2}$ the thermal noise power for the cGNSS-R case can be obtained from (A6):

(A7) $$P_{T_{c}}\left(\tau \right)=\frac{kT_{Nr}}{T_{c}}.$$

This expression demonstrates a reduction of the noise equivalent bandwidth due to the coherent integration process.

The correlation of $\rho_{0}\left(\tau \right)$ (deterministic) is:

(A8) $$E\left\{\rho_{0}\left(\tau_{1}\right)\rho_{0}^{*}\left(\tau_{2}\right)\right\}=P_{\mathrm{coh}}\left(\tau \right)=\frac{E_{T}G_{R}D_{R}^{2}\left(0\right)\Lambda^{2}\left[\tau_{1}-\tau_{2}-\frac{R_{0,sp}+R_{sp}}{c}\right]{|S\left(0\right)|}^{2}\lambda^{2}}{{\left(4\pi \right)}^{2}{\left(R_{0,sp}+R_{sp}\right)}^{2}}{|r\left(\theta \right)|}^{2}e^{-4\kappa^{2}\sigma_{h}^{2}\cos^{2}\left(\theta_{inc}\right)},$$

where $G_{R}$ stands for the receiving antenna gain, and has assumed that the coherent reflected power does not vary with time. It also shows that this only exists when the signal correlation is maximal (for a determined value of *τ*). In other words, it is only defined for $\tau =\tau_{1}-\tau_{2}=\frac{R_{0,sp}+R_{sp}}{c}$.

The correlation of $n_{S}\left(t,\tau \right)$ has been studied in several works in the literature for the sea surface (in the absence of a coherent component) [6,7,8,9,23,36]. A dedicated study is required to analyze this term in detail. For instance, ref. [9] assumes that between 1 ms waveforms the correlation function is a Kronecker delta function. This fact occurs, for instance, with the thermal noise correlation function as $\gamma_{n_{Tc},n_{Tc}}\left(t_{1}-t_{2},\tau \right)$ sampled at $t_{1}-t_{2}\propto 1\mathrm{ms}$ is equivalent to a Kronecker delta function too. Herein, it has been decided to use a Gaussian correlation function whose correlation time is computed based on the Van Cittert-Zernike theorem [39]. Therefore:

(A9) $$\begin{array}{cc} E\{n_{S}\left(t_{1},\tau_{1}\right)n_{S}^{*}\left(t_{2},\tau_{2}\right)\}= & E\left\{\frac{1}{T_{c}}\int_{-\frac{T_{c}}{2}}^{+\frac{T_{c}}{2}}u_{r_{inc}}\left(t_{1}+t^{\prime }+\tau_{1}\right)a\left(t_{1}+t^{\prime }\right)dt^{\prime }\frac{1}{T_{c}}\int_{-\frac{T_{c}}{2}}^{+\frac{T_{c}}{2}}u_{r_{inc}}^{*}\left(t_{2}+t^{\prime \prime }+\tau_{2}\right)a\left(t_{2}+t^{\prime \prime }\right)dt^{\prime \prime }\right\}=\\ = & \frac{1}{T_{c}^{2}}\int_{-\frac{T_{c}}{2}}^{+\frac{T_{c}}{2}}dt^{\prime }\int_{-\frac{T_{c}}{2}}^{+\frac{T_{c}}{2}}dt^{\prime \prime }a\left(t_{1}+t^{\prime }\right)a\left(t_{2}+t^{\prime \prime }\right)E\left\{u_{r_{inc}}\left(t_{1}+t^{\prime }+\tau_{1}\right)u_{r_{inc}}^{*}\left(t_{2}+t^{\prime \prime }+\tau_{2}\right)\right\}. \end{array}$$

Taking into account that:

(A10) $$u_{r_{inc}}\left({\vec{R}}_{r},t\right)=\int D\left(\vec{r}\right)a\left[t-\left(R_{0}-R\right)/c\right]g_{inc}\left(\vec{r},t\right)d\vec{r},$$

then:

(A11) $$\begin{array}{cc} E\{n_{S}\left(t_{1},\tau_{1}\right)n_{S}^{*}\left(t_{2},\tau_{2}\right)\}= & \int \frac{1}{T_{c}^{2}}\int_{-\frac{T_{c}}{2}}^{+\frac{T_{c}}{2}}dt^{\prime }\int_{-\frac{T_{c}}{2}}^{+\frac{T_{c}}{2}}dt^{\prime \prime }a\left(t_{1}+t^{\prime }\right)a\left(t_{2}+t^{\prime \prime }\right)a\left(t_{1}+t^{\prime }+\tau_{1}-\left(R_{0}-R\right)/c\right)\times \\ & a\left(t_{2}+t^{\prime \prime }+\tau_{2}-\left(R_{0}-R\right)/c\right)\rho_{u_{r_{inc}},u_{r_{inc}}}\left(t_{1}-t_{2}+\tau_{1}-\tau_{2}+t^{\prime }-t^{\prime \prime }\right)d\vec{r}d{\vec{r}}^{\prime }, \end{array}$$

where it has been assumed that the surface remains frozen for the coherent integration time, and $\rho_{u_{r_{inc}},u_{r_{inc}}}\left(t_{1}-t_{2}+\tau_{1}-\tau_{2}+t^{\prime }-t^{\prime \prime }\right)$ is the correlation function of the $g_{inc}$ function. Then:

(A12) $$\begin{array}{cc} E\{n_{S}\left(t_{1},\tau_{1}\right)n_{S}^{*}\left(t_{2},\tau_{2}\right)\}= & \int \frac{1}{2T_{c}^{2}}\int_{-T_{c}}^{0}d\xi \rho_{u_{r_{inc}},u_{r_{inc}}}\left(t_{1}-t_{2}+\tau_{1}-\tau_{2}+\xi \right)\int_{-\xi -T_{c}}^{\xi +T_{c}}a\left(t_{1}+\frac{\eta }{2}+\frac{\xi }{2}\right)a\left(t_{2}+\frac{\eta }{2}-\frac{\xi }{2}\right)\times \\ & a\left(t_{1}+\frac{\eta }{2}+\frac{\xi }{2}+\tau_{1}-\left(R_{0}-R\right)/c\right)a\left(t_{2}+\frac{\eta }{2}-\frac{\xi }{2}+\tau_{2}-\left(R_{0}-R\right)/c\right)d\eta d\vec{r}d{\vec{r}}^{\prime }+\\ + & \frac{1}{2T_{c}^{2}}\int_{0}^{T_{c}}d\xi \rho_{u_{r_{inc}},u_{r_{inc}}}\left(t_{1}-t_{2}+\tau_{1}-\tau_{2}+\xi \right)\int_{\xi -T_{c}}^{-\xi +T_{c}}a\left(t_{1}+\frac{\eta }{2}+\frac{\xi }{2}\right)a\left(t_{2}+\frac{\eta }{2}-\frac{\xi }{2}\right)\times \\ & a\left(t_{1}+\frac{\eta }{2}+\frac{\xi }{2}+\tau_{1}-\left(R_{0}-R\right)/c\right)a\left(t_{2}+\frac{\eta }{2}-\frac{\xi }{2}+\tau_{2}-\left(R_{0}-R\right)/c\right)d\eta d\vec{r}d{\vec{r}}^{\prime }=\\ = & \int \frac{1}{T_{c}}\int_{-T_{c}}^{T_{c}}\rho_{u_{r_{inc}},u_{r_{inc}}}\left(t_{1}-t_{2}+\tau_{1}-\tau_{2}+\xi \right)\Lambda \left(\frac{\xi }{T_{c}}\right)R_{a,a}\left(\tau_{1}-\left(R_{0}-R\right)/c\right)\times \\ & R_{a,a}\left(\tau_{2}-\left(R_{0}-R\right)/c\right)d\xi d\vec{r}d{\vec{r}}^{\prime }. \end{array}$$

This equation can be rearranged into two different integrals as the convolution of two different functions, the surface correlation function and the Λ function. Then:

(A13) $$E\left\{n_{S}\left(t_{1},\tau_{1}\right)n_{S}^{*}\left(t_{2},\tau_{2}\right)\right\}=\frac{1}{T_{c}}\left(R_{u_{r_{inc}},u_{r_{inc}}}*\Lambda_{T_{c}}\right)\left(t_{1}-t_{2}+\tau_{1}-\tau_{2}\right),$$

where

(A14) $$R_{u_{r_{inc}},u_{r_{inc}}}\left(t_{1}-t_{2}+\tau_{1}-\tau_{2}\right)=\int R_{a,a}\left(\tau_{1}-\left(R_{0}-R\right)/c\right)R_{a,a}\left(\tau_{2}-\left(R_{0}-R\right)/c\right)\rho_{u_{r_{inc}},u_{r_{inc}}}\left(t_{1}-t_{2}+\tau_{1}-\tau_{2}\right)d\vec{r}d{\vec{r}}^{\prime },$$

and it stands for the surface correlation function. Assuming that $\tau_{1}=\tau_{2}=\left(R_{0}-R\right)/c$, then $R_{a,a}\left(\tau_{1}-\left(R_{0}-R\right)/c\right)$ is equal to one in the surface correlation integral, and therefore:

(A15) $$E\left\{n_{S}\left(t_{1},\tau \right)n_{S}^{*}\left(t_{2},\tau \right)\right\}=P_{\mathrm{incoh}}\left(\tau \right)\gamma_{s,s}\left(t_{1}-t_{2},\tau \right)=\frac{1}{T_{c}}\left(\rho_{u_{r_{inc}},u_{r_{inc}}}\left(t_{1}-t_{2}\right)*\Lambda \left(\frac{t_{1}-t_{2}}{T_{c}}\right)\right).$$

If a Gaussian correlation function is assumed, then:

(A16) $$\rho_{u_{r_{inc}},u_{r_{inc}}}\left(t_{1}-t_{2},\tau =0\right)=P_{\mathrm{incoh}}\left(\tau \right)e^{-{\left(\frac{t_{1}-t_{2}}{t_{c}}\right)}^{2}},$$

where $P_{\mathrm{incoh}}\left(\tau \right)$ is the incoherent received power at a given delay which is given by [18]

(A17) $$P_{\mathrm{incoh}}\left(\tau \right)=\frac{EIRP_{T}G_{R}}{{\left(4\pi \right)}^{2}}I_{A_{illpq}}\left(\tau \right),I_{A_{illpq}}\left(\tau \right)=\lambda^{2}\int_{A_{ill}}\frac{\sigma^{0}\left(\vec{\rho }\right)D_{T}^{2}\left(\vec{\rho }\right)D_{R}^{2}\left(\vec{\rho }\right)\Lambda^{2}\left(\tau ,\vec{\rho }\right){\left|S\left[\tau ,\vec{\rho }\right]\right|}^{2}}{4\pi R_{0}^{2}\left(\vec{\rho }\right)R^{2}\left(\vec{\rho }\right)}d^{2}\rho ,$$

and

(A18) $$t_{c}\approx 2\cdot \frac{\lambda }{2v_{r}}\sqrt{\frac{R}{c\tau_{chip}}}$$

where $v_{r}$ is the platform’s speed, and the initial 2 has been added because the surface is illuminated with a triangular pulse instead of a square one due to the ACF function shape of the satellite codes. A reference to compute the equivalent ACF for PRN codes different from the C/A is [16]. The $\gamma_{s,s}\left(t_{1}-t_{2},\tau =0\right)$ becomes:

(A19) $$\gamma_{s,s}\left(t_{1}-t_{2},\tau =0\right)=\frac{1}{T_{c}}\left(e^{{\left(\frac{t_{1}-t_{2}}{t_{c}}\right)}^{2}}*\Lambda \left(\frac{t_{1}-t_{2}}{T_{c}}\right)\right).$$

Finally, the $E\{n_{T,i}\left(t_{1},\tau \right)n_{T,i}^{*}\left(t_{2},\tau \right)\}$ breaks into different terms which will be computed in the following equations. So, the $n_{T,i}\left(t,\tau \right)$ is defined as follows:

(A20) $$n_{T,i}\left(t,\tau \right)=n_{T,c}\left(t,\tau \right)+\sqrt{\frac{1}{SNR_{d}}}\left(y_{u_{r},d_{t}}\left(t,\tau \right)+y_{r_{t},d_{t}}\left(t,\tau \right)\right)$$

Hence, taking into account that all the terms are mutually independent, and that their respective mean value is 0, it is obtained:

(A21) $$\begin{array}{cc} E\{n_{T,i}\left(t_{1},\tau_{1}\right)n_{T,i}^{*}\left(t_{2},\tau_{2}\right)\}= & E\{n_{T,c}\left(t_{1},\tau_{1}\right)n_{T,c}^{*}\left(t_{2},\tau_{2}\right)\}+\\ & +\frac{1}{SNR_{d}}\left(E\left\{y_{u_{r},d_{t}}\left(t_{1},\tau_{1}\right)y_{u_{r},d_{t}}^{*}\left(t_{2},\tau_{2}\right)\right\}+E\left\{y_{d_{t},r_{t}}\left(t_{1},\tau_{1}\right)y_{d_{t},r_{t}}^{*}\left(t_{2},\tau_{2}\right)\right\}\right), \end{array}$$

where:

(A22) $$E\left\{n_{T,c}\left(t_{1},\tau \right)n_{T,c}^{*}\left(t_{2},\tau \right)\right\}=\frac{kT_{Nr}}{T_{c}}\Lambda \left(\frac{t_{1}-t_{2}}{T_{c}}\right)R_{a,a}\left(\tau_{1}-\tau_{2}\right),$$

(A23) $$\begin{array}{cc} E\{y_{u_{r},d_{t}}\left(t_{1},\tau_{1}\right)y_{u_{r},d_{t}}^{*}\left(t_{2},\tau_{2}\right)\}= & \frac{1}{T_{c}^{2}}\int_{-\frac{T_{c}}{2}}^{+\frac{T_{c}}{2}}dt^{\prime }\int_{-\frac{T_{c}}{2}}^{+\frac{T_{c}}{2}}dt^{\prime \prime }E\left\{u_{r}\left(t_{1}+t^{\prime }+\tau_{1}\right)u_{r}^{*}\left(t_{2}+t^{\prime \prime }+\tau_{2}\right)\right\}\times \\ & E\{{\bar{n}}_{d_{t}}\left(t_{1}+t^{\prime }+\tau_{1}\right){\bar{n}}_{d_{t}}^{*}\left(t_{2}+t^{\prime \prime }+\tau_{2}\right)\}, \end{array}$$

where ${\bar{n}}_{d_{t}}$ is the normalized thermal noise power (recall that by definition the direct noise signal has been normalized by the power of the clean direct signal). Assuming the same band-limited noise properties as previously, Equation (A23) becomes:

(A24) $$\begin{array}{cc} E\{y_{u_{r},d_{t}}\left(t_{1},\tau_{1}\right)y_{u_{r},d_{t}}^{*}\left(t_{2},\tau_{2}\right)\}= & \frac{1}{BT_{c}^{2}}\int_{-\frac{T_{c}}{2}}^{+\frac{T_{c}}{2}}dt^{\prime }\int_{-\infty }^{+\infty }dt^{\prime \prime }E\left\{u_{r}\left(t_{1}+t^{\prime }+\tau_{1}\right)u_{r}^{*}\left(t_{2}+t^{\prime \prime }+\tau_{2}\right)\right\}\times \\ & \Pi \left(\frac{t^{\prime \prime }-T_{c}/2}{T_{c}/2}\right)\frac{\sin\left(\pi B(t_{1}+t^{\prime }\tau_{1}-t_{2}-t^{\prime \prime }-\tau_{2})\right)}{\pi (t_{1}+t^{\prime }\tau_{1}-t_{2}-t^{\prime \prime }-\tau_{2})}=\\ = & \frac{1}{BT_{c}^{2}}\int_{-\frac{T_{c}}{2}}^{+\frac{T_{c}}{2}}dt^{\prime }E\{|u_{r}\left(t_{1}+t^{\prime }+\tau_{1}\right){|}^{2}\}\Pi \left(\frac{t_{1}-t_{2}+\tau_{1}-\tau_{2}+t^{\prime }-T_{c}/2}{T_{c}/2}\right). \end{array}$$

The term $E\{|u_{r}\left(t_{1}+t^{\prime }+\tau \right){|}^{2}\}$ stands for the total reflected signal power $\left[P_{\mathrm{coh}}\left(\tau \right)+P_{\mathrm{incoh}}\left(\tau \right)\right]$, and due to stationarity it can be taken out of the integral. Therefore:

(A25) $$\begin{array}{cc} E\{y_{u_{r},d_{t}}\left(t_{1},\tau_{1}\right)y_{u_{r},d_{t}}^{*}\left(t_{2},\tau_{2}\right)\} & =\frac{P_{\mathrm{coh}}\left(\tau \right)+P_{\mathrm{incoh}}\left(\tau \right)}{BT_{c}^{2}}\int_{-\frac{T_{c}}{2}}^{+\frac{T_{c}}{2}}dt^{\prime }\Pi \left(\frac{t_{1}-t_{2}+\tau_{1}-\tau_{2}+t^{\prime }-T_{c}/2}{T_{c}/2}\right)=\\ & =\frac{1}{BT_{c}}\left(P_{\mathrm{coh}}\left(\tau \right)+P_{\mathrm{incoh}}\left(\tau \right)\right)\Lambda \left(\frac{t_{1}-t_{2}+\tau_{1}-\tau_{2}}{T_{c}}\right). \end{array}$$

If considering the same delay ($\tau_{1}=\tau_{2}$), then:

(A26) $$E\left\{y_{u_{r},t_{d}}\left(t_{1},\tau \right)y_{u_{r},t_{d}}^{*}\left(t_{2},\tau \right)\right\}=\frac{1}{BT_{c}}\left(P_{\mathrm{coh}}\left(\tau \right)+P_{\mathrm{incoh}}\left(\tau \right)\right)\Lambda \left(\frac{t_{1}-t_{2}}{T_{c}}\right).$$

The term $E\{y_{r_{t},d_{t}}\left(t_{1},\tau_{1}\right)y_{r_{t},d_{t}}^{*}\left(t_{2},\tau_{2}\right)\}$ is given by:

(A27) $$\begin{array}{cc} E\{y_{r_{t},d_{t}}\left(t_{1},\tau_{1}\right)y_{r_{t},d_{t}}^{*}\left(t_{2},\tau_{2}\right)\} & =\frac{1}{T_{c}^{2}}\int_{-\frac{T_{c}}{2}}^{+\frac{T_{c}}{2}}dt^{\prime }\int_{-\frac{T_{c}}{2}}^{+\frac{T_{c}}{2}}dt^{\prime \prime }E\left\{n_{r_{t}}\left(t_{1}+t^{\prime }+\tau_{1}\right)n_{r_{t}}^{*}\left(t_{2}+t^{\prime \prime }+\tau_{2}\right)\right\}\times \\ & E\{{\bar{n}}_{d_{t}}\left(t_{1}+t^{\prime }+\tau_{1}\right){\bar{n}}_{d_{t}}^{*}\left(t_{2}+t^{\prime \prime }+\tau_{2}\right)\}=\\ & =\frac{kT_{Nr}}{T_{c}}\frac{1}{BT_{c}}\int_{-\frac{T_{c}}{2}}^{+\frac{T_{c}}{2}}dt^{\prime }\int_{-\frac{T_{c}}{2}}^{+\frac{T_{c}}{2}}dt^{\prime \prime }\frac{\sin^{2}\left(\pi B(t_{1}+t^{\prime }+\tau_{1}-t_{2}-t^{\prime \prime }-\tau_{2})\right)}{{\left(\pi (t_{1}+t^{\prime }-t_{2}-t^{\prime \prime })\right)}^{2}}, \end{array}$$

and this integral can be solved by changing the variables, $\xi =t^{\prime }-t^{\prime \prime }$, $\eta =t^{\prime }+t^{\prime \prime }$, and considering that the Jacobian of the transformation is 1/2. Hence:

(A28) $$E\left\{y_{r_{t},d_{t}}\left(t_{1},\tau \right)y_{r_{t},d_{t}}^{*}\left(t_{2},\tau \right)\right\}=\frac{kT_{Nr}}{T_{c}}\frac{1}{B}\int_{-T_{c}}^{+T_{c}}\Lambda \left(\frac{x}{T_{c}}\right)\frac{\sin^{2}\left(\pi B(t_{1}-t_{2}+\tau_{1}-\tau_{2}+x)\right)}{{\left(\pi (t_{1}-t_{2}+\tau_{1}-\tau_{2}+x)\right)}^{2}}dx.$$

If $1/B\ll T_{c}$, which always occurs since there is a minimum of 3 orders of magnitude difference between them, the $sinc^{2}$ function is much more narrower than the Λ function. This results in being able to take the Λ function out of the integral. Therefore:

(A29) $$\begin{array}{cc} E\{y_{r_{t},d_{t}}\left(t_{1},\tau \right)y_{r_{t},d_{t}}^{*}\left(t_{2},\tau \right)\} & =\frac{kT_{Nr}}{T_{c}}\frac{1}{B}\Lambda \left(\frac{t_{1}-t_{2}+\tau_{1}-\tau_{2}}{T_{c}}\right)\int_{-T_{c}}^{+T_{c}}\frac{\sin^{2}\left(\pi B(t_{1}-t_{2}+\tau_{1}-\tau_{2}+x)\right)}{{\left(\pi (t_{1}-t_{2}+\tau_{1}-\tau_{2}+x)\right)}^{2}}dx=\\ & =\frac{kT_{Nr}}{T_{c}}\Lambda \left(\frac{t_{1}-t_{2}+\tau_{1}-\tau_{2}}{T_{c}}\right), \end{array}$$

where the symmetry property of the Λ function has been used. If, for all cases $\tau_{1}=\tau_{2}$, then the correlation function of the equivalent iGNSS-R thermal noise becomes:

(A30) $$E\left\{n_{T,i}\left(t_{1},\tau \right)n_{T,i}^{*}\left(t_{2},\tau \right)\right\}=\Lambda \left(\frac{t_{1}-t_{2}}{T_{c}}\right)\cdot \left(\frac{kT_{Nr}}{T_{c}}+\frac{1}{SNR_{d}}\left[\frac{1}{BT_{c}}\left(P_{\mathrm{coh}}\left(\tau \right)+P_{\mathrm{incoh}}\left(\tau \right)\right)+\frac{kT_{Nr}}{T_{c}}\right]\right),$$

and simplifying:

(A31) $$E\left\{n_{T,i}\left(t_{1},\tau \right)n_{T,i}^{*}\left(t_{2},\tau \right)\right\}=\Lambda \left(\frac{t_{1}-t_{2}}{T_{c}}\right)2\sigma_{t,c}^{2}\left(\tau \right)\cdot \left(1+\frac{1}{SNR_{d}}\left(SNR_{r}+1\right)\right),$$

where $SNR_{r}$ refers to the pre-correlation SNR for the reflected signal:

(A32) $$SNR_{r}=\frac{P_{\mathrm{coh}}\left(\tau \right)+P_{\mathrm{incoh}}\left(\tau \right)}{kT_{Nr}B},$$

and $\gamma_{n_{Ti},n_{Ti}}\left(t_{1}-t_{2},\tau \right)=\gamma_{n_{Tc},n_{Tc}}\left(t_{1}-t_{2},\tau \right)$.

## Appendix B. Fourth Order Correlation Functions

Assuming that the above mentioned processes are Gaussian processes, the fourth order correlation functions can be expressed as a function of the second order correlation functions [17]. Therefore the shape of the fourth order correlation functions is:

(B1) $$\Gamma_{x,x}\left(t_{1}-t_{2},\tau \right)={\left(2\sigma^{2}\left(\tau \right)\right)}^{2}\left(1+|\gamma_{x,x}\left(t_{1}-t_{2},\tau \right){|}^{2}\right).$$

Therefore:

(B2) $$E\left\{n_{S}\left(t_{1},\tau \right)n_{S}^{*}\left(t_{1},\tau \right)n_{S}\left(t_{2},\tau \right)n_{S}^{*}\left(t_{2},\tau \right)\right\}=P_{\mathrm{incoh}}^{2}\left(\tau \right)\left(1+{\left|\frac{1}{T_{c}}\left[e^{{\left(\frac{t_{1}-t_{2}}{t_{c}}\right)}^{2}}*\Lambda \left(\frac{t_{1}-t_{2}}{T_{c}}\right)\right]\right|}^{2}\right)=\Gamma_{S,S}\left(t_{1}-t_{2},\tau \right),$$

(B3) $$E\left\{n_{T,c}\left(t_{1},\tau \right)n_{T,c}^{*}\left(t_{1},\tau \right)n_{T,c}\left(t_{2},\tau \right)n_{T,c}^{*}\left(t_{2},\tau \right)\right\}=P_{T_{c}}^{2}\left(\tau \right)\left(1+{\left|\Lambda \left(\frac{t_{1}-t_{2}}{T_{c}}\right)\right|}^{2}\right)=\Gamma_{n_{Tc},n_{Tc}}\left(t_{1}-t_{2},\tau \right),$$

(B4) $$E\left\{n_{T,i}\left(t_{1},\tau \right)n_{T,i}^{*}\left(t_{1},\tau \right)n_{T,i}\left(t_{2},\tau \right)n_{T,i}^{*}\left(t_{2},\tau \right)\right\}=P_{T_{i}}^{2}\left(\tau \right)\left(1+{\left|\Lambda \left(\frac{t_{1}-t_{2}}{T_{c}}\right)\right|}^{2}\right)=\Gamma_{n_{Ti},n_{Ti}}\left(t_{1}-t_{2},\tau \right).$$

## Appendix C. Detectability Criteria for the Different Cases

This Appendix details the computation of the detectability criteria shown in Section 4 when no incoherent integration has been applied. To compute each detectability criterion the following assumptions must be considered.

For the cGNSS-R:

(C1) $$f_{S+N}=Y_{c}\left(t,\tau \right)={\left|y_{c}\left(t,\tau \right)\right|}^{2},$$

(C2) $$f_{N}={\left|n_{T,c}\left(t,\tau \right)\right|}^{2},$$

and for the iGNSS-R:

(C3) $$f_{S+N}=Y_{i}\left(t,\tau \right)={\left|y_{i}\left(t,\tau \right)\right|}^{2},$$

(C4) $$f_{N}=|n_{T,c}\left(t,\tau \right)+\sqrt{\frac{1}{SNR_{d}}}y_{r_{t},d_{t}}\left(t,\tau \right){|}^{2}.$$

Note that in the absence of signal power $y_{u_{r},d_{t}}\left(t,\tau \right)=0$, which is why it does not appear in the $f_{N}$ expression for the iGNSS-R.

### Appendix C.1. Derivation of d_c_

As it has been shown in Section 4, the detectability criterion $d_{c}$ is:

(C5) $$d_{c}=\frac{E\left\{f_{S+N}\right\}-E\left\{f_{N}\right\}}{\sqrt{E\left\{f_{N}^{2}\right\}-E{\left\{f_{N}\right\}}^{2}}}=\frac{P_{\mathrm{coh}}\left(\tau \right)+P_{\mathrm{incoh}}\left(\tau \right)+P_{T_{c}}\left(\tau \right)-P_{T_{c}}\left(\tau \right)}{\sqrt{2P_{T_{c}}^{2}\left(\tau \right)-P_{T_{c}}^{2}\left(\tau \right)}}=\frac{P_{\mathrm{coh}}\left(\tau \right)+P_{\mathrm{incoh}}\left(\tau \right)}{P_{T_{c}}\left(\tau \right)},$$

where:

(C6) $$\begin{array}{cc} Y_{c}\left(t,\tau \right) & =y_{c}\left(t,\tau \right){y_{c}\left(t,\tau \right)}^{*}={\left|\rho_{0}\left(t,\tau \right)\right|}^{2}+\rho_{0}\left(t,\tau \right)n_{S}^{*}\left(t,\tau \right)+\rho_{0}\left(t,\tau \right)n_{T,c}^{*}\left(t,\tau \right)+\rho_{0}^{*}\left(t,\tau \right)n_{S}\left(t,\tau \right)+\\ & +{\left|n_{S}\left(t,\tau \right)\right|}^{2}+n_{S}\left(t,\tau \right)n_{T,c}^{*}\left(t,\tau \right)+\rho_{0}^{*}\left(t,\tau \right)n_{T,c}\left(t,\tau \right)+n_{T,c}\left(t,\tau \right)n_{S}^{*}\left(t,\tau \right)+{\left|n_{T,c}\left(t,\tau \right)\right|}^{2}, \end{array}$$

(C7) $$\begin{array}{cc} E\{f_{S+N}\} & =E\{{\left|\rho_{0}\left(t,\tau \right)\right|}^{2}+{\left|n_{S}\left(t,\tau \right)\right|}^{2}+{\left|n_{T,c}\left(t,\tau \right)\right|}^{2}\}=\\ & =P_{\mathrm{coh}}\left(\tau \right)+P_{\mathrm{incoh}}\left(\tau \right)\gamma_{s,s}\left(0,\tau \right)+P_{T_{c}}\left(\tau \right)\gamma_{n_{Tc},n_{Tc}}\left(0,\tau \right)=\\ & =P_{\mathrm{coh}}\left(\tau \right)+P_{\mathrm{incoh}}\left(\tau \right)+P_{T_{c}}\left(\tau \right), \end{array}$$

(C8) $$E\left\{f_{N}\right\}=E\left\{{\left|n_{T,c}\left(t,\tau \right)\right|}^{2}\right\}=P_{T_{c}}\left(\tau \right)\gamma_{n_{Tc},n_{Tc}}\left(0,\tau \right)=P_{T_{c}}\left(\tau \right),$$

and using the properties of Gaussian processes:

(C9) $$E\left\{{f_{N}}^{2}\right\}=E\left\{{\left|n_{T,c}\left(t,\tau \right)\right|}^{4}\right\}=\Gamma_{n_{Tc},n_{Tc}}\left(0,\tau \right)=P_{T_{c}}^{2}\left(\tau \right)\left(1+|\gamma_{n_{Tc},n_{Tc}}{\left(0,\tau \right)|}^{2}\right)=2P_{T_{c}}^{2}\left(\tau \right).$$

### Appendix C.2. Derivation of $d_{c}^{\prime }$

As it has been shown in Section 4, the detectability criterion $d_{c}^{\prime }$ is:

(C10) $$\begin{array}{cc} d_{c}^{\prime } & =\frac{E\left\{f_{S+N}\right\}-E\left\{f_{N}\right\}}{\sqrt{E\left\{f_{S+N}^{2}\right\}-E{\left\{f_{S+N}\right\}}^{2}}}=\\ & =\frac{P_{\mathrm{coh}}\left(\tau \right)+P_{\mathrm{incoh}}\left(\tau \right)+P_{T_{c}}\left(\tau \right)-P_{T_{c}}\left(\tau \right)}{\sqrt{2{\left(P_{\mathrm{coh}}\left(\tau \right)+P_{\mathrm{incoh}}\left(\tau \right)+P_{T_{c}}\left(\tau \right)\right)}^{2}-P_{\mathrm{coh}}^{2}\left(\tau \right)-{\left(P_{\mathrm{coh}}\left(\tau \right)+P_{\mathrm{incoh}}\left(\tau \right)+P_{T_{c}}\left(\tau \right)\right)}^{2}}}=\\ & =\frac{P_{\mathrm{coh}}\left(\tau \right)+P_{\mathrm{incoh}}\left(\tau \right)}{\sqrt{{\left(P_{\mathrm{coh}}\left(\tau \right)+P_{\mathrm{incoh}}\left(\tau \right)+P_{T_{c}}\left(\tau \right)\right)}^{2}-P_{\mathrm{coh}}^{2}\left(\tau \right)}}=\frac{1}{\sqrt{{\left(1+\frac{1}{{\mathrm{SNR}}_{{\mathrm{TH}}_{c}}}\right)}^{2}-{\left(1-\frac{1}{{\mathrm{SNR}}_{\mathrm{SP}}}\right)}^{2}}}, \end{array}$$

where:

(C11) $$\begin{array}{cc} E\{{f_{S+N}}^{2}\}= & E\left\{Y_{c}^{2}\left(t,\tau \right)\right\}=E\left\{Y_{c}\left(t,\tau \right)Y_{c}\left(t,\tau \right)\right\}=E\left\{{\left|\rho_{0}\left(t,\tau \right)\right|}^{4}\right\}+E\left\{{\left|n_{S}\left(t,\tau \right)\right|}^{4}\right\}+E\left\{{\left|n_{T,c}\left(t,\tau \right)\right|}^{4}\right\}+\\ & +4E\left\{{\left|\rho_{0}\left(t,\tau \right)\right|}^{2}{\left|n_{S}\left(t,\tau \right)\right|}^{2}\right\}+4E\left\{{\left|\rho_{0}\left(t,\tau \right)\right|}^{2}{\left|n_{T,c}\left(t,\tau \right)\right|}^{2}\right\}+4E\left\{{\left|n_{S}\left(t,\tau \right)\right|}^{2}{\left|n_{T,c}\left(t,\tau \right)\right|}^{2}\right\}=\\ = & 2{\left(P_{\mathrm{coh}}\left(\tau \right)+P_{\mathrm{incoh}}\left(\tau \right)+P_{T_{c}}\left(\tau \right)\right)}^{2}-P_{\mathrm{coh}}^{2}\left(\tau \right), \end{array}$$

(C12) $${\mathrm{SNR}}_{{\mathrm{TH}}_{c}}=\frac{P_{\mathrm{coh}}\left(\tau \right)+P_{\mathrm{incoh}}\left(\tau \right)}{P_{T_{c}}\left(\tau \right)},$$

(C13) $${\mathrm{SNR}}_{\mathrm{SP}}=\frac{P_{\mathrm{coh}}\left(\tau \right)+P_{\mathrm{incoh}}\left(\tau \right)}{P_{\mathrm{incoh}}\left(\tau \right)}.$$

### Appendix C.3. Derivation of d_i_

As it has been shown in Section 4, the detectability criterion $d_{i}$ is:

(C14) $$\begin{array}{cc} d_{i} & =\frac{E\left\{f_{S+N}\right\}-E\left\{f_{N}\right\}}{\sqrt{E\left\{f_{N}^{2}\right\}-E{\left\{f_{N}\right\}}^{2}}}=\\ & =\frac{P_{\mathrm{coh}}\left(\tau \right)+P_{\mathrm{incoh}}\left(\tau \right)+P_{T_{c}}\left(\tau \right)\left(1+\frac{1}{SNR_{d}}\left(SNR_{r}+1\right)\right)-P_{T_{c}}\left(\tau \right)\left(1+\frac{1}{SNR_{d}}\right)}{\sqrt{2P_{T_{c}\left(\tau \right)}^{2}{\left(1+\frac{1}{SNR_{d}}\right)}^{2}-P_{T_{c}}^{2}\left(\tau \right){\left(1+\frac{1}{SNR_{d}}\right)}^{2}}}=\\ & =\frac{P_{\mathrm{coh}}\left(\tau \right)+P_{\mathrm{incoh}}\left(\tau \right)+P_{T_{c}}\left(\tau \right)\frac{SNR_{r}}{SNR_{d}}}{P_{T_{c}\left(\tau \right)}\left(1+\frac{1}{SNR_{d}}\right)}=\frac{1+\frac{1}{d_{c}}\frac{SNR_{r}}{SNR_{d}}}{\frac{1}{d_{c}}\left(1+\frac{1}{SNR_{d}}\right)}\approx d_{c}\frac{1}{1+\frac{1}{SNR_{d}}}, \end{array}$$

where:

(C15) $$\begin{array}{cc} Y_{i}\left(t,\tau \right) & =y_{i}\left(t,\tau \right){y_{i}\left(t,\tau \right)}^{*}={|y_{c}\left(t,\tau \right)|}^{2}+\sqrt{\frac{1}{SNR_{d}}}y_{c}\left(t,\tau \right)y_{u_{r},d_{t}}^{*}\left(t,\tau \right)+\sqrt{\frac{1}{SNR_{d}}}y_{c}\left(t,\tau \right)y_{r_{t},d_{t}}^{*}\left(t,\tau \right)+\\ & +\sqrt{\frac{1}{SNR_{d}}}y_{u_{r},d_{t}}\left(t,\tau \right)y_{c}^{*}\left(t,\tau \right)+\frac{1}{SNR_{d}}{|y_{u_{r},d_{t}}\left(t,\tau \right)|}^{2}+\sqrt{\frac{1}{SNR_{d}}}y_{u_{r},d_{t}}\left(t,\tau \right)y_{r_{t},d_{t}}^{*}\left(t,\tau \right)+\\ & +\sqrt{\frac{1}{SNR_{d}}}y_{r_{t},d_{t}}\left(t,\tau \right)y_{c}^{*}\left(t,\tau \right)+\sqrt{\frac{1}{SNR_{d}}}y_{r_{t},d_{t}}\left(t,\tau \right)y_{u_{r},d_{t}}^{*}\left(t,\tau \right)+\frac{1}{SNR_{d}}{|y_{r_{t},d_{t}}\left(t,\tau \right)|}^{2}, \end{array}$$

(C16) $$\begin{array}{cc} E\{f_{S+N}\} & =E\left\{{|y_{c}\left(t,\tau \right)|}^{2}+\frac{1}{SNR_{d}}{|y_{u_{r},d_{t}}\left(t,\tau \right)|}^{2}+\frac{1}{SNR_{d}}{|y_{r_{t},d_{t}}\left(t,\tau \right)|}^{2}\right\}=\\ & =P_{\mathrm{coh}}\left(\tau \right)+P_{\mathrm{incoh}}\left(\tau \right)+P_{T_{c}}\left(\tau \right)\left(1+\frac{1}{SNR_{d}}\left(SNR_{r}+1\right)\right), \end{array}$$

(C17) $$E\left\{f_{N}\right\}=E\left\{|n_{T,c}\left(t,\tau \right)+\sqrt{\frac{1}{SNR_{d}}}y_{r_{t},d_{t}}\left(t,\tau \right){|}^{2}\right\}=P_{T_{c}}\left(\tau \right)\left(1+\frac{1}{SNR_{d}}\right),$$

and

(C18) $$\begin{array}{cc} E\{{f_{N}}^{2}\} & =E\left\{|n_{T,c}\left(t,\tau \right)+\sqrt{\frac{1}{SNR_{d}}}y_{r_{t},d_{t}}\left(t,\tau \right){|}^{4}\right\}=E\{(|n_{T,c}{\left(t,\tau \right)|}^{2}+\frac{1}{SNR_{d}}{\left|y_{r_{t},d_{t}}\left(t,\tau \right)\right|}^{2}+\\ & +\sqrt{\frac{1}{SNR_{d}}}n_{T,c}\left(t,\tau \right)y_{r_{t},d_{t}}^{*}\left(t,\tau \right)+\sqrt{\frac{1}{SNR_{d}}}n_{T,c}^{*}\left(t,\tau \right)y_{r_{t},d_{t}}\left(t,\tau \right))({\left|n_{T,c}\left(t,\tau \right)\right|}^{2}+\\ & +\frac{1}{SNR_{d}}{\left|y_{r_{t},d_{t}}\left(t,\tau \right)\right|}^{2}+\sqrt{\frac{1}{SNR_{d}}}n_{T,c}^{*}\left(t,\tau \right)y_{r_{t},d_{t}}\left(t,\tau \right)+\sqrt{\frac{1}{SNR_{d}}}n_{T,c}\left(t,\tau \right)y_{r_{t},d_{t}}^{*}\left(t,\tau \right))\}. \end{array}$$

To solve these moments, the results from Appendix B can be used. Therefore:

(C19) $$\Gamma_{n_{Tc},n_{Tc}}\left(t_{1}-t_{2},\tau \right)={\left(2\sigma_{t,c}^{2}\left(\tau \right)\right)}^{2}\left(1+|\gamma_{n_{Tc},n_{Tc}}\left(t_{1}-t_{2},\tau \right){|}^{2}\right)=P_{T_{c}}^{2}\left(\tau \right)\left(1+\Lambda^{2}\left(\frac{t_{1}-t_{2}}{T_{c}}\right)\right),$$

(C20) $$\Gamma_{y_{r_{t},d_{t}},y_{r_{t},d_{t}}}\left(t_{1}-t_{2},\tau \right)=\frac{P_{T_{c}}^{2}\left(\tau \right)}{SNR_{d}^{2}}\left(1+\Lambda^{2}\left(\frac{t_{1}-t_{2}}{T_{c}}\right)\right).$$

Hence:

(C21) $$\begin{array}{cc} E\{{f_{N}}^{2}\} & =E\{|n_{Tc}{\left(t,\tau \right)|}^{4}\}+\frac{4}{SNR_{d}}E\{|n_{T,c}{\left(t,\tau \right)|}^{2}|y_{r_{t},d_{t}}{\left(t,\tau \right)|}^{2}\}+\frac{1}{SNR_{d}^{2}}E\{|y_{r_{t},d_{t}}\left(t,\tau \right){|}^{4}\}=\\ & =2P_{T_{c}\left(\tau \right)}^{2}{\left(1+\frac{1}{SNR_{d}}\right)}^{2}. \end{array}$$

### Appendix C.4. Derivation of $d_{i}^{\prime }$

As it has been shown in Section 4, the detectability criterion $d_{i}^{\prime }$ is:

(C22) $$\begin{array}{cc} k_{i} & =\frac{E\left\{f_{S+N}\right\}-E\left\{f_{N}\right\}}{\sqrt{E\left\{f_{S+N}^{2}\right\}-E{\left\{f_{S+N}\right\}}^{2}}}=\\ & =\frac{P_{\mathrm{coh}}\left(\tau \right)+P_{\mathrm{incoh}}\left(\tau \right)+P_{T_{c}}\left(\tau \right)\frac{SNR_{r}}{SNR_{d}}}{\sqrt{2{\left(P_{\mathrm{coh}}\left(\tau \right)+P_{\mathrm{incoh}}\left(\tau \right)+P_{T_{i}}\left(\tau \right)\right)}^{2}-P_{\mathrm{coh}}^{2}\left(\tau \right)-{\left(P_{\mathrm{coh}}\left(\tau \right)+P_{\mathrm{incoh}}\left(\tau \right)+P_{T_{i}}\left(\tau \right)\right)}^{2}}}=\\ & =\frac{P_{\mathrm{coh}}\left(\tau \right)+P_{\mathrm{incoh}}\left(\tau \right)+P_{T_{c}}\left(\tau \right)\frac{SNR_{r}}{SNR_{d}}}{\sqrt{{\left(P_{\mathrm{coh}}\left(\tau \right)+P_{\mathrm{incoh}}\left(\tau \right)+P_{T_{i}}\left(\tau \right)\right)}^{2}-P_{\mathrm{coh}}^{2}\left(\tau \right)}}=\frac{1+\frac{1}{d_{c}}\frac{SNR_{r}}{SNR_{d}}}{\sqrt{{\left(1+\frac{1}{{\mathrm{SNR}}_{{\mathrm{TH}}_{i}}}\right)}^{2}-{\left(1-\frac{1}{{\mathrm{SNR}}_{\mathrm{SP}}}\right)}^{2}}}\approx \\ & \approx \frac{1}{\sqrt{{\left(1+\frac{1}{{\mathrm{SNR}}_{{\mathrm{TH}}_{i}}}\right)}^{2}-{\left(1-\frac{1}{{\mathrm{SNR}}_{\mathrm{SP}}}\right)}^{2}}}. \end{array}$$

In this case, the term $E\{{f_{S+N}}^{2}\}$ can be computed similarly to the previous computation of the $k_{c}^{\prime }$ parameter, but considering the noise term as $n_{T_{i}}\left(t,\tau \right)$ instead of $n_{T_{c}}\left(t,\tau \right)$. Therefore:

(C23) $$E\left\{{f_{S+N}}^{2}\right\}=2{\left(P_{\mathrm{coh}}\left(\tau \right)+P_{\mathrm{incoh}}\left(\tau \right)+P_{T_{i}}\left(\tau \right)\right)}^{2}-P_{\mathrm{coh}}^{2}\left(\tau \right),$$

where:

(C24) $$P_{T_{i}}\left(\tau \right)=P_{T_{c}}\left(\tau \right)\left(1+\frac{1}{SNR_{d}}\left(SNR_{r}+1\right)\right),$$

(C25) $${\mathrm{SNR}}_{{\mathrm{TH}}_{i}}=\frac{P_{\mathrm{coh}}\left(\tau \right)+P_{\mathrm{incoh}}\left(\tau \right)}{P_{T_{i}}\left(\tau \right)}=\frac{P_{\mathrm{coh}}\left(\tau \right)+P_{\mathrm{incoh}}\left(\tau \right)}{P_{T_{c}}\left(\tau \right)\left(1+\frac{1}{SNR_{d}}\left(SNR_{r}+1\right)\right)}=\frac{{\mathrm{SNR}}_{{\mathrm{TH}}_{c}}}{1+\frac{1}{SNR_{d}}\left(SNR_{r}+1\right)}.$$

## Appendix D. Detectability Criteria for the Different Cases after Non Coherent Integration

This Appendix details the computation of the detectability criteria shown in Section 5 when incoherent integration has been applied. To derive each detectability criterion the following assumptions must be considered.

For the cGNSS-R:

(D1) $$f_{S+N}=\frac{1}{T}\int_{0}^{T}Y_{c}\left(t+t^{\prime },\tau \right)dt^{\prime },$$

(D2) $$f_{N}=\frac{1}{T}\int_{0}^{T}{\left|n_{T,c}\left(t+t^{\prime },\tau \right)\right|}^{2}dt^{\prime },$$

and for the iGNSS-R:

(D3) $$f_{S+N}=\frac{1}{T}\int_{0}^{T}Y_{i}\left(t+t^{\prime },\tau \right)dt^{\prime },$$

(D4) $$f_{N}=\frac{1}{T}\int_{0}^{T}|n_{T,c}\left(t+t^{\prime },\tau \right)+\sqrt{\frac{1}{SNR_{d}}}y_{r_{t},d_{t}}\left(t+t^{\prime },\tau \right){|}^{2}dt^{\prime }.$$

### Appendix D.1. Derivation of d_nc_

As it has been shown in Section 5, $d_{nc}$ is given by:

(D5) $$\begin{array}{cc} d_{nc} & =\frac{E\left\{f_{S+N}\right\}-E\left\{f_{N}\right\}}{\sqrt{E\left\{f_{N}^{2}\right\}-E{\left\{f_{N}\right\}}^{2}}}=\frac{P_{\mathrm{coh}}\left(\tau \right)+P_{\mathrm{incoh}}\left(\tau \right)+P_{T_{c}}\left(\tau \right)-P_{T_{c}}\left(\tau \right)}{\sqrt{P_{T_{c}}^{2}\left(\tau \right)\left(1+{\bar{T}}_{n}\right)-P_{T_{c}}^{2}\left(\tau \right)}}=\\ & =\sqrt{\frac{3}{2}\frac{T}{T_{coh}}}\frac{P_{\mathrm{coh}}\left(\tau \right)+P_{\mathrm{incoh}}\left(\tau \right)}{P_{T_{c}}\left(\tau \right)}=\sqrt{\frac{3}{2}\frac{T}{T_{coh}}}d_{c}, \end{array}$$

where:

(D6) $$E\left\{f_{S+N}\right\}=E\left\{\frac{1}{T}\int_{0}^{T}Y_{c}\left(t+t^{\prime },\tau \right)dt^{\prime }\right\}=\frac{1}{T}\int_{0}^{T}E\left\{Y_{c}\left(t+t^{\prime },\tau \right)\right\}dt^{\prime }=P_{\mathrm{coh}}\left(\tau \right)+P_{\mathrm{incoh}}\left(\tau \right)+P_{T_{c}}\left(\tau \right),$$

(D7) $$E\left\{f_{N}\right\}=E\left\{\frac{1}{T}\int_{0}^{T}{\left|n_{T,c}\left(t+t^{\prime },\tau \right)\right|}^{2}dt^{\prime }\right\}=\frac{1}{T}\int_{0}^{T}E\{|n_{T,c}\left(t+t^{\prime },\tau \right){|}^{2}\}dt^{\prime }=P_{T_{c}}\left(\tau \right),$$

and using the properties of Gaussian processes:

(D8) $$\begin{array}{cc} E\{{f_{N}}^{2}\}= & E\left\{\frac{1}{T}\int_{0}^{T}|n_{T,c}\left(t+t^{\prime },\tau \right){|}^{2}dt^{\prime }\frac{1}{T}\int_{0}^{T}{\left|n_{T,c}\left(t+t^{\prime \prime },\tau \right)\right|}^{2}dt^{\prime \prime }\right\}=\\ = & \frac{1}{T^{2}}\int_{0}^{T}dt^{\prime }\int_{0}^{T}dt^{\prime \prime }E\{|n_{T,c}\left(t+t^{\prime },\tau \right){|}^{2}|n_{T,c}\left(t+t^{\prime \prime },\tau \right){|}^{2}\}=\frac{1}{T^{2}}\int_{0}^{T}dt^{\prime }\int_{0}^{T}dt^{\prime \prime }\Gamma_{n_{Tc},n_{Tc}}\left(t^{\prime }-t^{\prime \prime },\tau \right)=\\ = & \frac{1}{T^{2}}\int_{0}^{T}dt^{\prime }\int_{0}^{T}dt^{\prime \prime }P_{T_{c}}^{2}\left(\tau \right)\left(1+\Lambda^{2}\left(\frac{t^{\prime }-t^{\prime \prime }}{T_{coh}}\right)\right)=P_{T_{c}}^{2}\left(\tau \right)+\frac{P_{T_{c}}^{2}\left(\tau \right)}{T^{2}}\int_{0}^{T}dt^{\prime }\int_{0}^{T}dt^{\prime \prime }\Lambda^{2}\left(\frac{t^{\prime }-t^{\prime \prime }}{T_{coh}}\right)=\\ = & P_{T_{c}}^{2}\left(\tau \right)+\frac{P_{T_{c}}^{2}\left(\tau \right)}{T}\int_{-T}^{T}\Lambda \left(\frac{\xi }{T}\right)\Lambda^{2}\left(\frac{\xi }{T_{coh}}\right)d\xi =P_{T_{c}}^{2}\left(\tau \right)\left(1+{\bar{T}}_{n}\right)=P_{T_{c}}^{2}\left(\tau \right)\left(1+\frac{2}{3}\frac{T_{coh}}{T}\right). \end{array}$$

### Appendix D.2. Derivation of $d_{nc}^{\prime }$

As it has been shown in Section 5, $d_{nc}^{\prime }$ is given by:

(D9) $$\begin{array}{cc} d_{nc}^{\prime } & =\frac{E\left\{f_{S+N}\right\}-E\left\{f_{N}\right\}}{\sqrt{E\left\{f_{S+N}^{2}\right\}-E{\left\{f_{S+N}\right\}}^{2}}}=\\ & =\frac{P_{\mathrm{coh}}\left(\tau \right)+P_{\mathrm{incoh}}\left(\tau \right)}{\sqrt{2{\bar{t}}_{s}P_{\mathrm{coh}}\left(\tau \right)P_{\mathrm{incoh}}\left(\tau \right)+2{\bar{t}}_{n}P_{\mathrm{coh}}\left(\tau \right)P_{T_{c}}\left(\tau \right)+2{\bar{t}}_{s}{\bar{t}}_{n}P_{\mathrm{incoh}}\left(\tau \right)P_{T_{c}}\left(\tau \right)+{\bar{T}}_{n}P_{T_{c}}^{2}\left(\tau \right)+{\bar{T}}_{s}P_{\mathrm{incoh}}^{2}\left(\tau \right)}}, \end{array}$$

where:

(D10) $$\begin{array}{cc} E\{{f_{S+N}}^{2}\} & =E\left\{\frac{1}{T}\int_{0}^{T}Y_{c}\left(t+t^{\prime },\tau \right)dt^{\prime }\frac{1}{T}\int_{0}^{T}Y_{c}\left(t+t^{\prime \prime },\tau \right)dt^{\prime \prime }\right\}=\\ & =\frac{1}{T^{2}}\int_{0}^{T}dt^{\prime }\int_{0}^{T}dt^{\prime \prime }E\left\{Y_{c}\left(t+t^{\prime },\tau \right)Y_{c}\left(t+t^{\prime \prime },\tau \right)\right\}= \end{array}$$

(D11) $$\begin{array}{cc} E\{{f_{S+N}}^{2}\}= & P_{\mathrm{coh}}^{2}\left(\tau \right)+2P_{\mathrm{coh}}\left(\tau \right)P_{\mathrm{incoh}}\left(\tau \right)+2P_{\mathrm{coh}}\left(\tau \right)P_{T_{c}}\left(\tau \right)+2P_{\mathrm{incoh}}\left(\tau \right)P_{T_{c}}\left(\tau \right)+\\ & +\frac{1}{T^{2}}\int_{0}^{T}\int_{0}^{T}2P_{\mathrm{coh}}\left(\tau \right)P_{\mathrm{incoh}}\left(\tau \right)\gamma_{s,s}\left(t^{\prime }-t^{\prime \prime },\tau \right)+2P_{\mathrm{coh}}\left(\tau \right)P_{T_{c}}\left(\tau \right)\gamma_{n_{Tc},n_{Tc}}\left(t^{\prime }-t^{\prime \prime },\tau \right)+\\ & +2P_{\mathrm{incoh}}\left(\tau \right)\gamma_{s,s}\left(t^{\prime }-t^{\prime \prime },\tau \right)P_{T_{c}}\left(\tau \right)\gamma_{n_{Tc},n_{Tc}}\left(t^{\prime }-t^{\prime \prime },\tau \right)+\Gamma_{s,s}\left(t^{\prime }-t^{\prime \prime },\tau \right)+\Gamma_{n_{Tc},n_{Tc}}\left(t^{\prime }-t^{\prime \prime },\tau \right)dt^{\prime }dt^{\prime \prime }= \end{array}$$

(D12) $$\begin{array}{cc} E\{{f_{S+N}}^{2}\}= & P_{\mathrm{coh}}^{2}\left(\tau \right)+2P_{\mathrm{coh}}\left(\tau \right)P_{\mathrm{incoh}}\left(\tau \right)+2P_{\mathrm{coh}}\left(\tau \right)P_{T_{c}}\left(\tau \right)+2P_{\mathrm{incoh}}\left(\tau \right)P_{T_{c}}\left(\tau \right)+\\ & +\frac{1}{T}\int_{-T}^{T}\Lambda \left(\frac{\xi }{T}\right)(2P_{\mathrm{coh}}\left(\tau \right)P_{\mathrm{incoh}}\left(\tau \right)\gamma_{s,s}\left(\xi ,\tau \right)+2P_{\mathrm{coh}}\left(\tau \right)P_{T_{c}}\left(\tau \right)\gamma_{n_{Tc},n_{Tc}}\left(\xi ,\tau \right)+\\ & +2P_{\mathrm{incoh}}\left(\tau \right)\gamma_{s,s}\left(\xi ,\tau \right)P_{T_{c}}\left(\tau \right)\gamma_{n_{Tc},n_{Tc}}\left(\xi ,\tau \right)+\Gamma_{s,s}\left(\xi ,\tau \right)+\Gamma_{n_{Tc},n_{Tc}}\left(\xi ,\tau \right))dt^{\prime }dt^{\prime \prime }= \end{array}$$

(D13) $$\begin{array}{cc} E\{{f_{S+N}}^{2}\}= & P_{\mathrm{coh}}^{2}\left(\tau \right)+2\left(1+{\bar{t}}_{s}\right)P_{\mathrm{coh}}\left(\tau \right)P_{\mathrm{incoh}}\left(\tau \right)+2\left(1+{\bar{t}}_{n}\right)P_{\mathrm{coh}}\left(\tau \right)P_{T_{c}}\left(\tau \right)+\\ & +2\left(1+{\bar{t}}_{s}{\bar{t}}_{n}\right)P_{\mathrm{incoh}}\left(\tau \right)P_{T_{c}}\left(\tau \right)+P_{T_{c}}^{2}\left(\tau \right)\left(1+{\bar{T}}_{n}\right)+P_{\mathrm{incoh}}^{2}\left(\tau \right)\left(1+{\bar{T}}_{s}\right), \end{array}$$

which uses the time definitions shown in Equation (31a)–(31d).

### Appendix D.3. Derivation of d_ni_

As it has been shown in Section 5, $d_{ni}$ is given by:

(D14) $$\begin{array}{cc} d_{ni}= & \frac{E\left\{f_{S+N}\right\}-E\left\{f_{N}\right\}}{\sqrt{E\left\{f_{N}^{2}\right\}-E{\left\{f_{N}\right\}}^{2}}}=\frac{P_{\mathrm{coh}}\left(\tau \right)+P_{\mathrm{incoh}}\left(\tau \right)+P_{T_{i}}\left(\tau \right)-P_{T_{c}}\left(\tau \right)\left(1+\frac{1}{SNR_{d}}\right)}{\sqrt{P_{T_{c}}^{2}\left(\tau \right)\left(1+{\bar{T}}_{n}\right){\left(1+\frac{1}{SNR_{d}}\right)}^{2}-P_{T_{c}}^{2}\left(\tau \right){\left(1+\frac{1}{SNR_{d}}\right)}^{2}}}=\\ = & \sqrt{\frac{3}{2}\frac{T}{T_{coh}}}\frac{P_{\mathrm{coh}}\left(\tau \right)+P_{\mathrm{incoh}}\left(\tau \right)+P_{T_{c}}\left(\tau \right)\frac{SNR_{r}}{SNR_{d}}}{P_{T_{c}}\left(\tau \right)\left(1+\frac{1}{SNR_{d}}\right)}=d_{nc}\frac{1}{1+\frac{1}{SNR_{d}}}+\sqrt{\frac{3}{2}\frac{T}{T_{coh}}}\frac{SNR_{r}}{SNR_{d}+1}, \end{array}$$

where:

(D15) $$E\left\{f_{S+N}\right\}=E\left\{\frac{1}{T}\int_{0}^{T}Y_{i}\left(t+t^{\prime },\tau \right)dt^{\prime }\right\}=\frac{1}{T}\int_{0}^{T}E\left\{Y_{i}\left(t+t^{\prime },\tau \right)\right\}dt^{\prime }=P_{\mathrm{coh}}\left(\tau \right)+P_{\mathrm{incoh}}\left(\tau \right)+P_{T_{i}}\left(\tau \right),$$

(D16) $$\begin{array}{cc} E\{f_{N}\} & =E\left\{\frac{1}{T}\int_{0}^{T}|n_{T,c}\left(t+t^{\prime },\tau \right)+\sqrt{\frac{1}{SNR_{d}}}y_{r_{t},d_{t}}\left(t+t^{\prime },\tau \right){|}^{2}dt^{\prime }\right\}=\\ & =\frac{1}{T}\int_{0}^{T}E\{|n_{T,c}\left(t+t^{\prime },\tau \right){|}^{2}\}dt^{\prime }+\frac{1}{SNR_{d}}\frac{1}{T}\int_{0}^{T}E\{|y_{r_{t},d_{t}}\left(t+t^{\prime },\tau \right){|}^{2}\}dt^{\prime }\\ & =P_{T_{c}}\left(\tau \right)\left(1+\frac{1}{SNR_{d}}\right), \end{array}$$

and using the properties of Gaussian processes, we obtain:

(D17) $$\begin{array}{cc} E\{{f_{N}}^{2}\}= & E\{\frac{1}{T}\int_{0}^{T}|n_{T,c}\left(t+t^{\prime },\tau \right)+\sqrt{\frac{1}{SNR_{d}}}y_{r_{t},d_{t}}\left(t+t^{\prime },\tau \right){|}^{2}dt^{\prime }\times \\ & \frac{1}{T}\int_{0}^{T}|n_{T,c}\left(t+t^{\prime \prime },\tau \right)+\sqrt{\frac{1}{SNR_{d}}}y_{r_{t},d_{t}}\left(t+t^{\prime \prime },\tau \right){|}^{2}dt^{\prime \prime }\}= \end{array}$$

(D18) $$\begin{array}{cc} E\{{f_{N}}^{2}\}= & \frac{1}{T^{2}}\int_{0}^{T}\int_{0}^{T}E\{|n_{T,c}\left(t+t^{\prime },\tau \right){|}^{2}|n_{T,c}\left(t+t^{\prime \prime },\tau \right){|}^{2}\}+\\ & +\frac{1}{SNR_{d}}E\{|n_{T,c}\left(t+t^{\prime },\tau \right){|}^{2}|y_{r_{t},d_{t}}\left(t+t^{\prime \prime },\tau \right){|}^{2}\}+\\ & +\frac{1}{SNR_{d}}E\left\{n_{T,c}\left(t+t^{\prime },\tau \right)n_{T,c}^{*}\left(t+t^{\prime \prime },\tau \right)y_{r_{t},d_{t}}\left(t+t^{\prime },\tau \right)y_{r_{t},d_{t}}^{*}\left(t+t^{\prime \prime },\tau \right)\right\}+\\ & +\frac{1}{SNR_{d}}E\left\{n_{T,c}\left(t+t^{\prime \prime },\tau \right)n_{T,c}^{*}\left(t+t^{\prime },\tau \right)y_{r_{t},d_{t}}\left(t+t^{\prime \prime },\tau \right)y_{r_{t},d_{t}}^{*}\left(t+t^{\prime },\tau \right)\right\}+\\ & +\frac{1}{SNR_{d}}E\{|n_{T,c}\left(t+t^{\prime \prime },\tau \right){|}^{2}|y_{r_{t},d_{t}}\left(t+t^{\prime },\tau \right){|}^{2}\}+\\ & +\frac{1}{SNR_{d}^{2}}E\{|y_{r_{t},d_{t}}\left(t+t^{\prime },\tau \right){|}^{2}|y_{r_{t},d_{t}}\left(t+t^{\prime \prime },\tau \right){|}^{2}\}dt^{\prime }dt^{\prime \prime }= \end{array}$$

(D19) $$\begin{array}{cc} E\{{f_{N}}^{2}\}= & \frac{1}{T^{2}}\int_{0}^{T}\int_{0}^{T}\Gamma_{n_{Tc},n_{Tc}}\left(t^{\prime }-t^{\prime \prime },\tau \right)+\frac{2P_{T_{c}}^{2}\left(\tau \right)}{SNR_{d}}\gamma_{n_{Tc},n_{Tc}}\left(0,\tau \right)\gamma_{y_{r_{t},d_{t}},y_{r_{t},d_{t}}}\left(0,\tau \right)+\\ & +\Gamma_{y_{r_{t},d_{t}},y_{r_{t},d_{t}}}\left(t^{\prime }-t^{\prime \prime },\tau \right)+\frac{2P_{T_{c}}^{2}\left(\tau \right)}{SNR_{d}}\gamma_{n_{Tc},n_{Tc}}\left(t^{\prime }-t^{\prime \prime },\tau \right)\gamma_{y_{r_{t},d_{t}},y_{r_{t},d_{t}}}\left(t^{\prime }-t^{\prime \prime },\tau \right)dt^{\prime }dt^{\prime \prime }=\\ = & P_{T_{c}}^{2}\left(\tau \right)\left(1+{\bar{T}}_{n}\right){\left(1+\frac{1}{SNR_{d}}\right)}^{2}. \end{array}$$

### Appendix D.4. Derivation of $d_{ni}^{\prime }$

As it has been shown in Section 5, $d_{ni}^{\prime }$ is given by:

(D20) $$\begin{array}{cc} d_{ni}^{\prime } & =\frac{E\left\{f_{S+N}\right\}-E\left\{f_{N}\right\}}{\sqrt{E\left\{f_{S+N}^{2}\right\}-E{\left\{f_{S+N}\right\}}^{2}}}=\\ & =\frac{P_{\mathrm{coh}}\left(\tau \right)+P_{\mathrm{incoh}}\left(\tau \right)+P_{T_{c}}\left(\tau \right)\frac{SNR_{r}}{SNR_{d}}}{\sqrt{2{\bar{t}}_{s}P_{\mathrm{coh}}\left(\tau \right)P_{\mathrm{incoh}}\left(\tau \right)+2{\bar{t}}_{n}P_{\mathrm{coh}}\left(\tau \right)P_{T_{i}}\left(\tau \right)+2{\bar{t}}_{s}{\bar{t}}_{n}P_{\mathrm{incoh}}\left(\tau \right)P_{T_{i}}\left(\tau \right)+{\bar{T}}_{n}P_{T_{i}}^{2}\left(\tau \right)+{\bar{T}}_{s}P_{\mathrm{incoh}}^{2}\left(\tau \right)}}, \end{array}$$

where:

(D21) $$\begin{array}{cc} E\{{f_{S+N}}^{2}\}= & P_{\mathrm{coh}}^{2}\left(\tau \right)+2\left(1+{\bar{t}}_{s}\right)P_{\mathrm{coh}}\left(\tau \right)P_{\mathrm{incoh}}\left(\tau \right)+2\left(1+{\bar{t}}_{n}\right)P_{\mathrm{coh}}\left(\tau \right)P_{T_{i}}\left(\tau \right)+\\ & +2\left(1+{\bar{t}}_{s}{\bar{t}}_{n}\right)P_{\mathrm{incoh}}\left(\tau \right)P_{T_{i}}\left(\tau \right)+P_{T_{i}}^{2}\left(\tau \right)\left(1+{\bar{T}}_{n}\right)+P_{\mathrm{incoh}}^{2}\left(\tau \right)\left(1+{\bar{T}}_{s}\right), \end{array}$$

which is obtained similarly to the case when $d_{nc}^{\prime }$ was computed, but substituting $P_{T_{c}}\left(\tau \right)$ by $P_{T_{i}}\left(\tau \right)$, which can be done because they have the same statistics and correlation functions, and only the scaling factor must be taken into account.
